# Organic cation transporter 1 (OCT1) modulates multiple cardiometabolic traits through effects on hepatic thiamine content

**DOI:** 10.1371/journal.pbio.2002907

**Published:** 2018-04-16

**Authors:** Xiaomin Liang, Sook Wah Yee, Huan-Chieh Chien, Eugene C. Chen, Qi Luo, Ling Zou, Meiling Piao, Arias Mifune, Ligong Chen, Meredith E. Calvert, Sarah King, Frode Norheim, Janna Abad, Ronald M. Krauss, Kathleen M. Giacomini

**Affiliations:** 1 Department of Bioengineering and Therapeutic Sciences, University of California, San Francisco, San Francisco, California, United States of America; 2 Department of Pharmacology and Pharmaceutical Sciences, School of Medicine, Tsinghua University, Beijing, China; 3 The Gladstone Institutes, Histology and Light Microscopy Core, San Francisco, California, United States of America; 4 Children's Hospital Oakland Research Institute, Oakland, California, United States of America; 5 Department of Medicine/Division of Cardiology, David Geffen School of Medicine, University of California, Los Angeles, Los Angeles, California, United States of America; 6 Institute for Human Genetics, University of California, San Francisco, San Francisco, California, United States of America; Wellcome Trust Sanger Institute, United Kingdom of Great Britain and Northern Ireland

## Abstract

A constellation of metabolic disorders, including obesity, dysregulated lipids, and elevations in blood glucose levels, has been associated with cardiovascular disease and diabetes. Analysis of data from recently published genome-wide association studies (GWAS) demonstrated that reduced-function polymorphisms in the organic cation transporter, OCT1 (*SLC22A1*), are significantly associated with higher total cholesterol, low-density lipoprotein (LDL) cholesterol, and triglyceride (TG) levels and an increased risk for type 2 diabetes mellitus, yet the mechanism linking OCT1 to these metabolic traits remains puzzling. Here, we show that OCT1, widely characterized as a drug transporter, plays a key role in modulating hepatic glucose and lipid metabolism, potentially by mediating thiamine (vitamin B1) uptake and hence its levels in the liver. Deletion of *Oct1* in mice resulted in reduced activity of thiamine-dependent enzymes, including pyruvate dehydrogenase (PDH), which disrupted the hepatic glucose–fatty acid cycle and shifted the source of energy production from glucose to fatty acids, leading to a reduction in glucose utilization, increased gluconeogenesis, and altered lipid metabolism. In turn, these effects resulted in increased total body adiposity and systemic levels of glucose and lipids. Importantly, wild-type mice on thiamine deficient diets (TDs) exhibited impaired glucose metabolism that phenocopied *Oct1* deficient mice. Collectively, our study reveals a critical role of hepatic thiamine deficiency through *OCT1* deficiency in promoting the metabolic inflexibility that leads to the pathogenesis of cardiometabolic disease.

## Author summary

The liver is the major organ for glucose and lipid metabolism; impairment in liver energy metabolism is often found in metabolic disorders. Traditionally, excesses in macronutrients (fat and glucose) are linked to the development of metabolic disorders. Our study provides evidence that imbalances in a micronutrient, vitamin B1 (thiamine), can serve as an etiological cause of lipid and glucose disorders and implicates the organic cation transporter, OCT1, in these disorders. OCT1 is a key determinant of thiamine levels in the liver. In humans, reduced-function polymorphisms of OCT1 significantly associate with high LDL cholesterol levels. Using Oct1 knockout mice, we show that reduced OCT1-mediated thiamine uptake in the liver leads to reduced levels of TPP—the active metabolite of thiamine—and decreased activity of key TPP-dependent enzymes. As a result, a shift from glucose to fatty acid oxidation occurs, leading to imbalances in key metabolic intermediates, alterations in metabolic flux pathways, and disruptions of various metabolic regulatory mechanisms. The extensive characterization of Oct1 knockout mice provides evidence for the molecular mechanisms responsible for various metabolic traits and indicates an important role for imbalances in micronutrients in cardiometabolic disorders.

## Introduction

Hepatic energy metabolism is a major determinant of systemic glucose and lipid levels as well as total body adiposity, which in turn are key risk factors for cardiovascular and metabolic diseases [1, 2]. Genome-wide association studies (GWAS) have provided a wealth of information on the genes and pathways involved in hepatic energy metabolism, including apolipoprotein E (*APOE*), proprotein convertase subtilisin/kexin type 9 (*PCSK9*), and low-density lipoprotein receptor (LDLR) [3–5]. In follow-up studies in cells and in preclinical animal models, most of these genes have been linked mechanistically to lipid metabolism [6]. In contrast, the mechanisms responsible for the genome-wide–level significant association of *SLC22A1* (encoding the organic cation transporter, OCT1) with total and low-density lipoprotein (LDL) cholesterol [3] remains unexplored.

In humans, the *OCT1* gene is highly polymorphic. A number of reduced-function variants with high prevalence in European populations have been characterized [7–9]. In particular, 40% of Caucasians carry one and 9% carry two reduced-function OCT1 variants [7, 8]. OCT1, which is highly expressed in the liver, has been widely characterized as a drug uptake transporter. Reduced-function polymorphisms of OCT1 have been associated with changes in the pharmacokinetics and pharmacodynamics of several drugs, including the opiate receptor agonist, morphine, and the anti-diabetic drug, metformin [10–12]. Recently, GWAS and fine mapping analysis showed that OCT1 functional variants are associated with acylcarnitine levels through efflux mechanism [13].

Previously, through metabolomic studies in *Oct1*^*-/-*^ mice and in cells overexpressing human *OCT1*, our laboratory identified thiamine, vitamin B1, as a major endogenous substrate for OCT1, and *Oct1* knockout mice were shown to exhibit hepatic thiamine deficiency [14]. Although systemic thiamine deficiency is well known to cause nerve damage and lead to beriberi and Wernicke-Korsakoff syndrome [15, 16], the pathophysiologic effects of thiamine deficiency in the liver are not understood. Thiamine pyrophosphate (TPP), the active metabolite of thiamine, is an essential cofactor for several metabolic enzymes, including pyruvate dehydrogenase (PDH), α-ketoglutarate dehydrogenase (α-KGDH), and transketolase (TK), which have fundamental roles in regulating cellular energy metabolism [15]. In particular, in 1963 Randle proposed that PDH acts as a key metabolic switch in the glucose–fatty acid cycle, which underlies the metabolic disturbance of diabetes. Under the theory of substrate competition between glucose and fatty acids, an increase in fatty acid oxidation and a reduction in glycolytic flux result in a critical imbalance in energy metabolism in tissues. As noted by Randle, regulation of PDH activity greatly influences selection of fuel source [17, 18]. Failure to flexibly adjust the choice of fuel (e.g., fatty acids or glucose) for metabolic energy production has recently been proposed to underlie metabolic inflexibility and lead to the pathogenesis associated with metabolic disorders [19]. Metabolic inflexibility and indeed metabolic syndrome have been linked to an excess of macronutrients (e.g., carbohydrates or fat); however, the role of micronutrients such as thiamine in metabolic syndrome has been largely ignored. Although many reports have identified a high prevalence of thiamine deficiency in patients with diabetes or obesity [20–23] and a beneficial effect of thiamine supplementation in these patient populations [24–26], the molecular mechanisms contributing to thiamine-associated metabolic disturbance are unknown.

Here, we hypothesize that reduced OCT1 function or reduced dietary thiamine intake leading to decreases in hepatic thiamine levels modulates the activity of multiple enzymes and the levels of key metabolites involved in glucose and lipid metabolism. These effects result in dyslipidemias, increases in circulating glucose levels, and peripheral adiposity. Through extensive experiments in *Oct1*^*-/-*^ mice, our data show that *Oct1* deficiency results in substantial changes in hepatic energy metabolism, i.e., reduction in glucose utilization, increased gluconeogenesis, and alterations in lipid metabolism. Similarly, feeding wild-type mice a thiamine deficient diet (TD) results in comparable effects on hepatic energy metabolism. Taken together, our studies suggest that hepatic thiamine deficiency, through deletion of *Oct1* in mice, results in the development of metabolic inflexibility. Our studies provide a mechanistic explanation for the striking metabolic findings in large-scale human genetic studies, demonstrating that common OCT1 reduced-function polymorphisms are associated with dyslipidemias, obesity, and increased risk for type 2 diabetes.

## Results

### OCT1 reduced-function variants are strongly associated with human lipid levels

**Fig 1:**
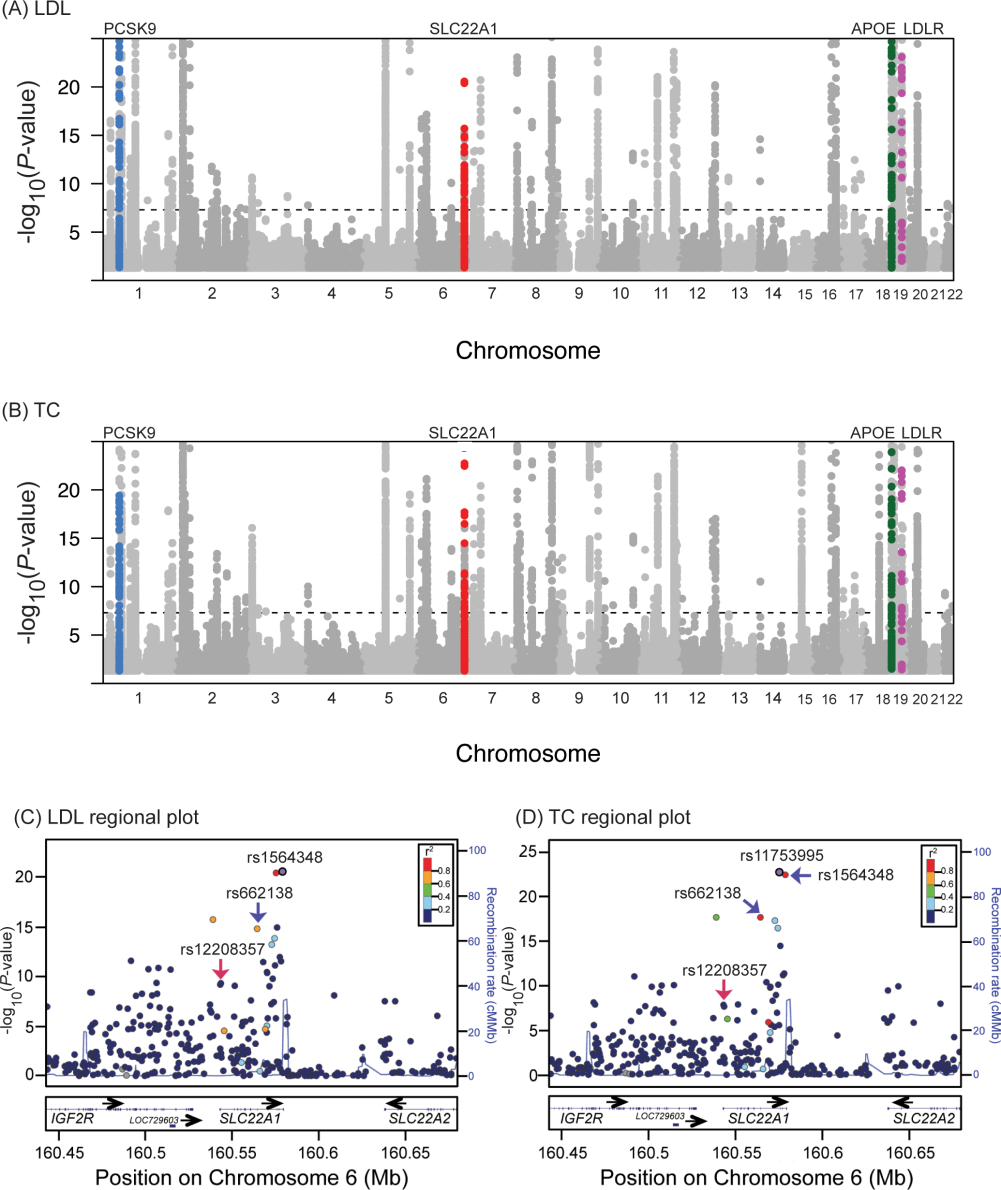
Manhattan plots and regional plots of the *SLC22A1* locus associated with lipid levels. Manhattan plots for (A) LDL cholesterol levels and (B) total cholesterol (−log10 P) in up to 188,577 individuals with European ancestries. The data are plotted using the results available from the Global Lipids Genetics Consortium, http://csg.sph.umich.edu/abecasis/public/lipids2013/ [3]. Only the SNPs with *p*-value ranges from 0.05 to 1 × 10^−25^ are plotted in (A) and (B). S1 Fig shows the Manhattan plots for all SNPs. *APOE*, *PCSK9*, and LDLR are among the genes previously known to associate with lipid levels, as highlighted in (A) and (B). Over 100 loci were associated with lipids at *p* < 5 × 10^−8^, including SLC22A1, which is the top locus in chromosome 6. The regional plots of the *SLC22A1* locus for (C) LDL cholesterol levels and (D) total cholesterol. SNPs are plotted by position on chromosome 6 (hg19) against association with meta-analysis of (C) LDL cholesterol levels and (D) total cholesterol in up to 188,577 individuals. The plots show that rs1564348 and rs11753995 (purple circles) are the top signals for (C) LDL cholesterol (*p* = 2.8 × 10^−21^) and (D) total cholesterol (*p* = 1.8 × 10^−23^), respectively. Both SNPs have strong linkage disequilibrium with the SLC22A1-420 deletion (rs202220802) (r^2^ = 0.78, D′ = 0.99) (http://archive.broadinstitute.org/mammals/haploreg/haploreg.php). The red arrow points to a nonsynonymous SNP, rs12208357 (SLC22A1-R61C), which is associated with (C) LDL cholesterol (*p* = 6.6 × 10^−10^) and with (D) total cholesterol (*p* = 1.3 × 10^−8^). Blue arrows point to an intronic SNP in *SLC22A1*, rs662138, which is included in many genome-wide genotyping platforms and also has strong linkage disequilibrium with the SLC22A1-420 deletion (rs202220802) (r^2^ = 0.78, D′ = 0.99). The associations of rs662138 with other traits are shown in Table 1. Estimated recombination rates (cM/Mb) are plotted in a blue line to reflect the local linkage disequilibrium structure. The SNPs surrounding the most significant SNP, (C) rs1564348 and (D) rs11753995, are color coded to reflect their linkage disequilibrium with other SNPs in the locus, based on pairwise r^2^ values from the HapMap CEU data. Genes, the position of exons, and the direction of transcription from the UCSC Genome Browser are noted. APOE, apolipoprotein E; CEU, Utah residents with Northern and Western European ancestry from the CEPH collection; *IGF2R*, insulin like growth factor 2 receptor; LDL, low-density lipoprotein; LDLR, low-density lipoprotein receptor; *LOC729603*, non-coding RNA; *PCSK9*, proprotein convertase subtilisin/kexin type 9; rs, reference single nucleotide polymorphisms (SNPs); *SLC*, Solute Carrier; TC, total cholesterol; UCSC, University of California, Santa Cruz.

The GWAS Catalog, database of Genotypes and Phenotypes (dbGAP) Association Results Browser, and Genome-Wide Repository of Associations Between SNPs and Phenotypes (GRASP) identified two major phenotypes (total cholesterol and LDL cholesterol levels) that were associated with genetic variants in *SLC22A1* (*OCT1*) (Fig 1 and S1A and S1B Fig). In particular, rs1564348 and rs11753995 were associated with LDL cholesterol (*p* = 2.8 × 10^−21^) and total cholesterol (*p* = 1.8 × 10^−23^), respectively (Fig 1 and Table 1). Using HaploReg v4.1 to obtain linkage disequilibrium information from 1000 Genomes Project, we noted that these two SNPs are in linkage with the OCT1 with methinone420 deletion (420Del), a common genetic variant in OCT1 that shows reduced uptake and altered kinetics of its substrates. Thus, the results suggest that reduced OCT1 function is significantly associated with higher total cholesterol and higher LDL levels. The GRASP database identified other phenotypes with significant, but weaker, *p*-values, relevant to glucose traits and coronary artery disease. Recent results from the UK Biobank cohort (http://geneatlas.roslin.ed.ac.uk/), available in the Gene ATLAS database and from the Global Lipids Genetic Consortium, are also included in Table 1. As shown, the reduced-function OCT1 nonsynonymous variants, OCT1-R61C, OCT1-G401S, OCT1-420Del, and OCT1-G465R, were significantly associated with high total cholesterol, LDL cholesterol, and/or TG levels in at least one study (Table 1). In addition, two of the missense OCT1 variants, OCT1-P341L and OCT1-V408M, which are associated with lower SLC22A1 expression levels in several tissues [13, 27, 28], were also associated with higher cholesterol levels in at least one study. The OCT1 nonsynonymous variants in Table 1, except OCT1-P341L, are not in linkage disequilibrium (r^2^ < 0.1) with SNPs in lipoprotein(a) (LPA) and lipoprotein(a) like 2 (LPAL2) genes (a known locus for plasma lipoprotein levels) [29–31] (S1C Fig), indicating that OCT1 constitutes an independent locus for association with plasma lipids, which was also recently shown in other studies [32, 33]. Notably, the effect size of the OCT1 variants for associations with lipids traits are small; thus, larger sample sizes are needed for genome-wide level significance (*p* < 5 × 10^−8^) (Table 1). In the Type 2 Diabetes Knowledge Portal, weaker but significant associations (*p* < 0.05) between OCT1 reduced-function variants and higher 2-hour glucose levels, higher fasting insulin levels, increased risk for type 2 diabetes, increased risk for coronary artery disease, and higher BMI were cataloged (Table 1). We performed burden test analysis using the data available in the portal. Interestingly, in the analysis, in which we included possibly or probably deleterious missense or protein truncating variants of OCT1, we observed strong associations of the reduced-function OCT1 variants with increased body weight (*p* = 0.0002–0.0005, beta = 0.23–0.3). When we performed a similar burden test analysis with type 2 diabetes, the significance was weaker and the results were only significant when we included only protein truncating variants of *OCT1* (*p* = 0.015, odds ratio = 2.10).

**Table 1:** Summary of the association of reduced-function OCT1 variants with various phenotypes through examination of publicly available datasets. Associations with *p* < 5 × 10^−8^ are in bold.

SNP	Amino Acid Change (Major Allele > Minor Allele); Allele Frequency^#^	Functional Effect	Clinical Phenotype	Results Associated with Minor Allele	*p*-value, Beta Coefficient, or Odds Ratio (Sample Size^##^)	References
rs12208357	R61C (C > T); 6%	Reduced uptake of thiamine and other substrates	Total cholesterol	Higher total cholesterol	*p* = 1.3 × 10^−8^ beta = 0.06 *N* = 92,398	[3, 34], also see footnotes ^a^^,^ ^b^
			LDL cholesterol	Higher LDL cholesterol	*p* = 6.6 × 10^−10^ beta = 0.07 *N* = 82,922	
			Triglycerides	Higher triglycerides	*p* = 0.0005 beta = 0.035 *N* = 86,546	
			Total cholesterol	Higher total cholesterol	*p* = 5.2 × 10^−21^ beta = 0.05 N = 311,483	[33], also see footnote ^c^
			LDL cholesterol	Higher LDL cholesterol	*p* = 6.4 × 10^−26^ beta = 0.06 *N* = 287,807	
			Triglycerides	Higher triglycerides	*p* = 3.9 × 10^−9^ beta = 0.005 *N* = 297,502	
			HDL cholesterol	Lower HDL cholesterol	*p* = 7.5 × 10^−7^ beta = −0.03 *N* = 308,296	
			LDL cholesterol	Higher LDL cholesterol	*p* = 9.6 × 10^−7^ beta = 0.067 *N* = 38,733	[35]
			Total cholesterol	Higher total cholesterol	*p* = 8.5 × 10^−14^ beta = 0.09 *N* = 62,085	[36]
			Total cholesterol	Higher total cholesterol	*p* = 0.01 beta = 0.05 *N* = 21,488	[37]
			LDL cholesterol	Higher LDL cholesterol	*p* = 0.01 beta = 0.05 *N* = 21,556	
			Triglycerides	Higher triglycerides	*p* = 0.02 beta = 0.05 *N* = 21,542	
rs683369	F160L (C > G); 21%	Small reduction in uptake of thiamine and other substrates. This SNP is in linkage disequilibrium to R61C with r^2^ = 0.2 and D′ = 1.	Total cholesterol	Higher total cholesterol	*p* = 6.3 × 10^−7^ beta = 0.02 *N* = 186,282	[3, 34], also see footnotes ^a^^,^ ^b^
			LDL cholesterol	Higher LDL cholesterol	*p* = 1.1 × 10^−6^ beta = 0.02 *N* = 172,142	
			Triglycerides	Higher triglycerides	*p* = 0.0088 beta = 0.012 *N* = 176,886	
			Total cholesterol	Higher total cholesterol	*p* = 1.6 × 10^−7^ beta = 0.02 *N* = 319,677	[33], also see footnote ^c^
			LDL cholesterol	Higher LDL cholesterol	*p* = 7.1 × 10^−11^ beta = 0.02 *N* = 295,826	
			Triglycerides	Higher triglycerides	*p* = 0.00029 beta = 0.01 *N* = 305,699	
			HDL cholesterol	Lower HDL cholesterol	*p* = 0.00035 beta = −0.01 *N* = 316,391	
			Total cholesterol	Higher total cholesterol	*p* = 0.0005 beta = 0.05 *N* = 21,489	[37]
			LDL cholesterol	Higher LDL cholesterol	*p* = 0.003 beta = 0.04 *N* = 21,557	
			Triglycerides	Higher triglycerides	*p* = 0.004 beta = 0.04 *N* = 21,543	
rs2282143	P341L (C > T); 1%	Minor allele is associated with lower expression levels of *SLC22A1* (GTEx^d^). This SNP is in linkage disequilibirum to missense LPA variant, Il891M, with r^2^ = 0.54 and D′ = 0.78.	Total cholesterol	Higher total cholesterol	*p* = 1.3 × 10^−8^ beta = 0.06 *N* = 92,398	[3, 34], also see footnote ^a^^,^ ^b^
			LDL cholesterol	Higher LDL cholesterol	*p* = 6.6 × 10^−10^ beta = 0.07 *N* = 82,922	
			Triglycerides	Higher triglycerides	*p* = 0.0005 beta = 0.035 *N* = 86,546	
			Total cholesterol	Higher total cholesterol	*p* = 1.7 × 10^−8^ beta = 0.05 *N* = 268,509	[33], also see footnote ^c^
			LDL cholesterol	Higher LDL cholesterol	*p* = 4.2 × 10^−11^ beta = 0.06 *N* = 247,909	
			Triglycerides	Higher triglycerides	*p* = 0.6 beta = 0.005 *N* = 255,115	
			HDL cholesterol	Lower HDL cholesterol	*p* = 0.15 beta = −0.01 *N* = 265,165	
			LDL cholesterol	Higher LDL cholesterol	*p* = 3.0 × 10^−9^ beta = 0.18 *N* = 38,733	[35]
rs34130495	G401S (G > A); 2%	Reduced uptake of thiamine and other substrates	Total cholesterol	Higher total cholesterol	*p* = 8.5 × 10^−7^ beta = 0.04 *N* = 309,101	[33], also see footnote ^c^
			LDL cholesterol	Higher LDL cholesterol	*p* = 6.8 × 10^−7^ beta = 0.04 *N* = 285,310	
			Triglycerides	Higher triglycerides	*p* = 6.3 × 10^−5^ beta = 0.04 *N* = 295,116	
			HDL cholesterol	Lower HDL cholesterol	*p* = 0.03 beta = −0.02 *N* = 305,872	
			Total cholesterol	Higher total cholesterol	*p* = 0.39 beta = 0.04 *N* = 21,489	[37]
			LDL cholesterol	Higher LDL cholesterol	*p* = 0.72 beta = 0.01 *N* = 21,557	
			Triglycerides	Higher triglycerides	*p* = 0.16 beta = 0.06 *N* = 21,543	
			Cholesterol	Higher cholesterol	*p* = 0.8 beta = 0.0004 *N* = 50,423	UK Biobank GeneATLAS^e^
			Disorders of lipoprotein metabolism and other lipidaemias	Increased risk	*p* = 0.6 beta = 0.0009 *N* = 38,897	UK Biobank GeneATLAS^e^
rs628031	V408M (G > A); 41%	Minor allele, A, is associated with lower expression of levels of S*LC22A1* (GTEx Portal^d^;[13, 28])	Total cholesterol	Higher total cholesterol	*p* = 3.8 × 10^−5^ beta = 0.02 *N* = 172,038	[3, 34], also see footnotes ^a^^,^ ^b^
			LDL cholesterol	Higher LDL cholesterol	*p* = 2.2 × 10^−6^ beta = 0.02 *N* = 160,099	
			Triglycerides	Higher triglycerides	*p* = 0.0025 beta = 0.01 *N* = 164,016	
			Total cholesterol	Higher total cholesterol	*p* = 0.0004 beta = 0.009 *N* = 319,677	[33], also see footnote ^c^
			LDL cholesterol	Higher LDL cholesterol	*p* = 1.4 × 10^−6^ beta = 0.01 *N* = 295,826	
			Triglycerides	Higher triglycerides	*p* = 0.07 beta = 0.005 *N* = 305,699	
			HDL cholesterol	Lower HDL cholesterol	*p* = 0.02 beta = −0.006 *N* = 316,391	
			Total cholesterol	Higher total cholesterol	*p* = 0.004 beta = 0.03 *N* = 17,830	[37]
			LDL cholesterol	Higher LDL cholesterol	*p* = 0.0006 beta = 0.04 *N* = 17,898	
			Triglycerides	Higher triglycerides	*p* = 0.05 beta = 0.02 *N* = 17,884	
rs662138, rs1564348	LD to 420del (r^2^ = 0.8, D′ = 1) (rs202220802); 18%	Reduced uptake of thiamine and other substrates	Total cholesterol	Higher total cholesterol	*p* = 2.0 × 10^−18^ beta = 0.04 *N* = 179,248	[3, 34], also see footnote ^a^^,^ ^b^
			LDL cholesterol	Higher LDL cholesterol	*p* = 1.7 × 10^−15^ beta = 0.04 *N* = 165,133	
			Triglycerides	Higher triglycerides	*p* = 0.0033 beta = 0.014 *N* = 169,786	
			Total cholesterol	Higher total cholesterol	*p* = 3.5 × 10^−37^ beta = 0.04 *N* = 319,677	[33], also see footnote ^c^
			LDL cholesterol	Higher LDL cholesterol	*p* = 2.1 × 10^−38^ beta = 0.05 *N* = 295,826	
			Triglycerides	Higher triglycerides	*p* = 2.8 × 10^−8^ beta = 0.02 *N* = 305,699	
			HDL cholesterol	Lower HDL cholesterol	*p* = 0.42 beta = −0.003 *N* = 316,391	
			Total cholesterol	Higher total cholesterol	*p* = 0.004 beta = 0.02 *N* = 58,325	[36]
			LDL cholesterol	Higher LDL cholesterol	*p* = 0.0025 beta = 0.03 *N* = 58,325	[36]
			Total cholesterol	Higher total cholesterol	*p* = 0.008 beta = 0.04 *N* = 21,486	[37]
			Triglycerides	Higher triglycerides	*p* = 0.04 beta = 0.03 *N* = 21,540	[37]
			Glucose	Higher 2-hour glucose	*p* = 0.028 beta = 0.058 *N* = 15,234	MAGIC^f^
			Body fat percentage	Higher body fat percentage	*p* = 0.0495 beta = 0.013 *N* = 89,449	GIANT^g^
			Coronary artery disease	Higher risk for developing coronary artery disease	*p* = 0.0016 OR = 1.04	Type 2 diabetes portal^h^
			Cholesterol	Higher cholesterol	*p* = 1.2 × 10^−13^ beta = 0.007 *N* = 50,423	UK Biobank GeneATLAS^e^
			Disorders of lipoprotein metabolism and other lipidaemias	Increase risk	*p* = 9.0 × 10^−11^ beta = 0.005 *N* = 38,897	UK Biobank GeneATLAS^e^
rs34059508	G465R (G > A); 2%	Reduced uptake of thiamine and other substrates	Total cholesterol	Higher total cholesterol	*p* = 1.8 × 10^−5^ beta = 0.04 *N* = 342,398	[38], also see footnote^c^
			LDL cholesterol	Higher LDL cholesterol	*p* = 2.9 × 10^−5^ beta = 0.04 *N* = 319,658	
			Triglycerides	Higher triglycerides	*p* = 0.17 beta = 0.03 *N* = 326,692	
			Total cholesterol	Higher total cholesterol	*p* = 0.84 beta = −0.009 *N* = 21,485	[37]
			LDL cholesterol	Higher LDL cholesterol	*p* = 0.57 beta = 0.03 *N* = 21,553	
			Triglycerides	Higher triglycerides	*p* = 0.72 beta = −0.02 *N* = 21,539	
			Triglycerides	Higher triglycerides	*p* = 0.0071 beta = 0.24 *N* = NA	Type 2 diabetes portal^h^
			Type 2 diabetes	Higher risk of diabetes	*p* = 0.03 OR = 1.43 *N* = GWAS SIGMA	Type 2 diabetes portal^h^
Burden testing of probably deleterious OCT1 missense or protein-truncating			BMI	Higher BMI	*p* = 0.0008–0.02 beta = 0.15–0.17	Type 2 diabetes portal^h^
Burden testing of probably deleterious OCT1 missense or protein-truncating			Glucose	Lower plasma glucose	*p* = 0.0012–0.000014 beta = −0.04–−0.12 95% CI: (−0.159–−0.0393)	Type 2 diabetes portal^h^

^#^Allele frequency: European ancestry from HapMap or 1000 Genomes Project (http://grch37.ensembl.org/Homo_sapiens/Info/Index).

^##^Sample size: information available only for data that were extracted from summary statistics of the GWAS.

Full datasets are available from

^a^http://csg.sph.umich.edu//abecasis/public/lipids2010/.

^b^http://csg.sph.umich.edu//abecasis/public/lipids2013/.

^c^http://csg.sph.umich.edu/abecasis/public/lipids2017/.

^d^https://www.gtexportal.org/home/.

^e^http://geneatlas.roslin.ed.ac.uk/.

^f^https://www.magicinvestigators.org/downloads/.

^g^http://portals.broadinstitute.org/collaboration/giant/index.php/GIANT_consortium_data_files.

^h^ http://www.type2diabetesgenetics.org/.

Abbreviations: A, adenine; C, cytosine; del, deletion; F160L, Phenylalanine to leucine in amino acid position 160; G, guanine; GWAS, genome-wide association study; G401S, Glycine to serine in amino acid position 401; G465R, Glycine to Arginine in amino acid position 465; HDL, high-density lipoprotein; I1891M, Isoleucine to methionine in amino acid position 1891; LD, linkage disequilibrium; LDL, low-density lipoprotein; LPA, lipoprotein(a); MAGIC, Meta-Analyses of Glucose and Insulin-related traits Consortium; NA, not available; OCT, organic cation transporter; OR, odds ratio; P341L, Proline to Leucine in amino acid position 341; SIGMA, Slim Initative for Genomic Medicine in the Americas; *SLC22A1*, solute carrier family 22 (organic cation transporter), member 1 (gene name for OCT1); T, thymine; V408M, Valine to methionine in amino acid position 408; 420del, OCT1 with methinone420 deletion.

### Deletion of *Oct1* altered hepatic and peripheral energy homeostasis

**Fig 2:**
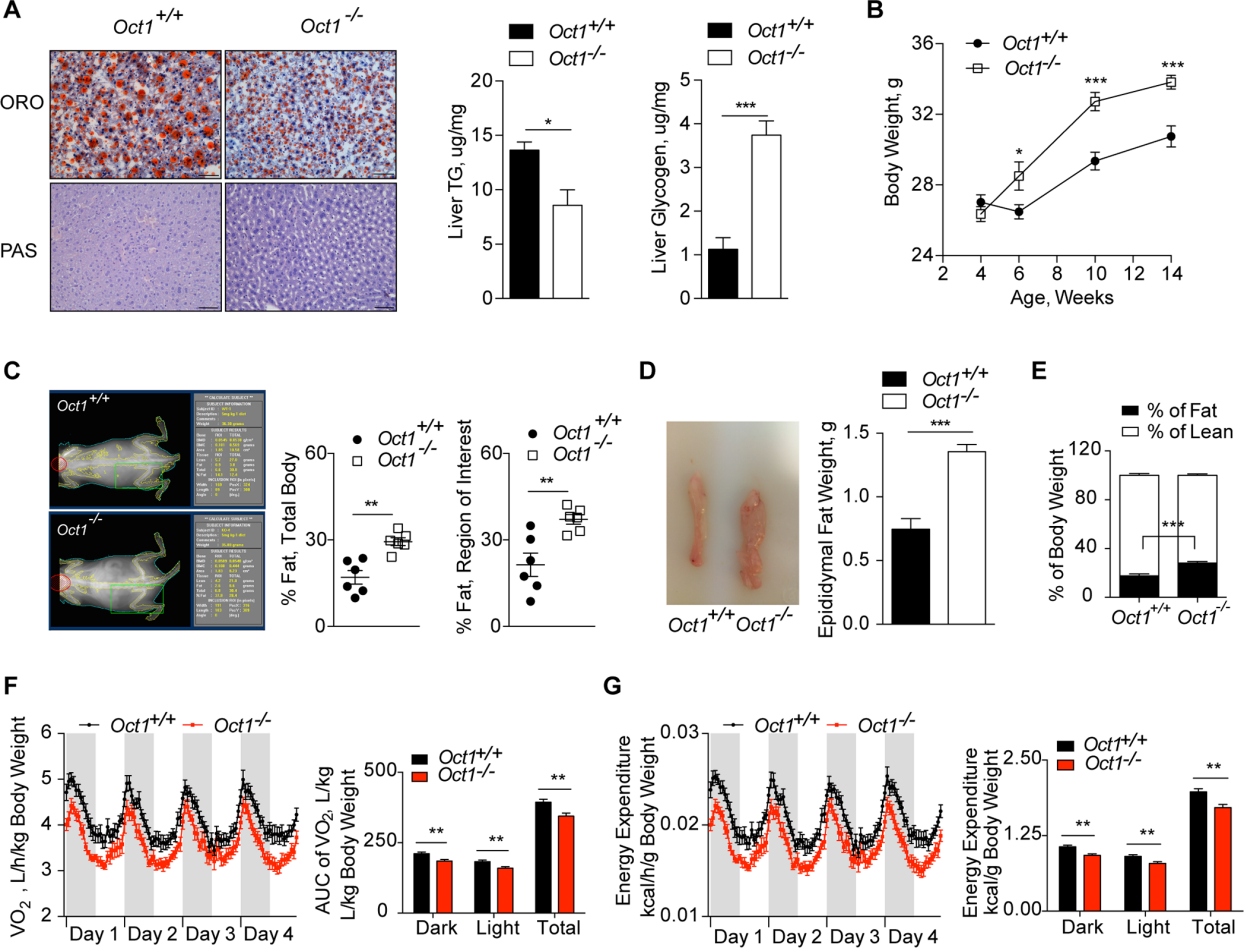
*Oct1* deletion altered energy homeostasis in vivo. Beginning at 5 weeks, mice were treated with a thiamine control diet. (A) Representative images of ORO and PAS staining of mouse livers (*n* = 3 per genotype); scale bars = 100 μm. Quantified hepatic triglyceride (*n* = 4 per genotype) and glycogen levels (*n* = 10 per genotype) in mice fasted 16 hours overnight. (B) Body weight of mice from ages 4 to 14 weeks (*n* = 12–24 per genotype at each time point). (C) Representative images for body composition measured by DEXA (*n* = 6 per genotype). Percent of total body fat and percent of fat in the region is indicated by the green square (*n* = 6 per genotype). (D) Representative images and weights of epididymal fat pads (*n* = 14 per genotype). (E) Body composition of 12-week-old mice measured by EchoMRI before CLAMS (*n* = 12 per genotype). (F) Respiratory O_2_ consumption normalized by total body weight for 96 hours and calculated AUC (*n* = 12 per genotype). (G) Energy expenditure normalized by total body weight for 96 hours and calculated AUC (*n* = 12 per genotype). Data shown are mean ± SEM. Data were analyzed by unpaired two-tailed Student *t* test; *p < 0.05, **p < 0.01, and ***p < 0.001. Underlying data are provided in S1 Data. AUC, area under the curve; CLAMS, comprehensive laboratory animal monitoring system; DEXA, dual-energy X-ray absorptiometry; *Oct1*, organic cation transporter 1; ORO, Oil Red-O; O_2_, oxygen; PAS, Periodic-Acid Schiff’s; TG, triglyceride; VO_2_, oxygen consumption.

Consistent with our previous studies, deletion of *Oct1* protected the mice from hepatic steatosis [14] (Fig 2A, S2A Fig). In this study, we observed that glycogen content was 3.3-fold greater in livers from *Oct1*^*-/-*^ mice compared to livers from *Oct1*^*+/+*^ mice after an overnight fast (Fig 2A and S2B Fig). Consistent with these results, hepatic glucose levels were 5.9-fold higher (*p* = 0.0006) in *Oct1*^*-/-*^ mice compared to *Oct1*^*+/+*^ mice (S2C Fig). Significantly greater body weights were observed for *Oct1*^*-/-*^ mice compared to their wild-type counterparts, starting at the age of 6 weeks (Fig 2B and S2D Fig). Body composition also differed, with dual-energy X-ray absorptiometry (DEXA) scans showing a higher percent of body fat in *Oct1*^*-/-*^ compared to *Oct1*^*+/+*^ mice (*p* = 0.001) (Fig 2C). Consistent with the greater proportion of body fat, *Oct1*^*-/-*^ mice had greater epididymal fat pad weights and reduced liver weight compared to *Oct1*^*+/+*^ mice (*p* < 0.0001) (Fig 2D and S2E Fig).

To further assess the potential mechanism leading to increased weight gain in *Oct1*^*-/-*^ mice, we analyzed energy expenditure, food intake, and activity by the comprehensive laboratory animal monitoring system (CLAMS). Before placing the mice into the CLAMS, the body composition of all mice was measured by EchoMRI. As shown in Fig 2E, *Oct1*^*-/-*^ mice had greater fat and lower lean mass in comparison to *Oct1*^*+/+*^ mice (*p* < 0.0001). When normalized to total body weight, *Oct1*^*-/-*^ mice had significantly lower respiratory oxygen (O_2_) consumption and energy expenditure (Fig 2F and 2G), indicating lower metabolic rates of *Oct1*^*-/-*^ mice in comparison to *Oct1*^*+/+*^ mice. These data are consistent with the lower lean mass of the *Oct1*^*-/-*^ mice compared to *Oct1*^*+/+*^ mice, because lean mass contributes more to energy expenditure than more inert tissue, such as adipose tissue [39, 40]. In fact, no differences in respiratory O_2_ consumption or energy expenditure normalized to lean mass were observed between *Oct1*^*+/+*^ and *Oct1*^*-/-*^ mice (S2F and S2G Fig). Thus, the differences in metabolic rate between *Oct1*^*+/+*^ and *Oct1*^*-/-*^ mice appear to be due to significant differences in body composition. Additionally, our *Oct1*^*-/-*^ mice had no difference in activity but had slightly lower food intake and respiratory exchange ratio (RER) during the dark cycle compared to *Oct1*^*+/+*^ mice (S2H–S2J Fig). There were no deleterious effects of *Oct1* deficiency on hepatic function and, in fact, some of the liver function tests improved in the *Oct1* knockout mice in comparison to wild-type mice (S2K Fig). There were no major differences in the expression levels of thiamine transporters (*Slc19a2* and *Slc19a3*) in the liver. In contrast, levels of organic cation transporter, *Oct2*, which also transports thiamine, were increased, albeit the expression levels of *Oct2* in the liver were extremely low relative to *Oct1* and *Slc19a2* (S2L Fig). Collectively, our data suggest that *Oct1* deletion had a significant effect on hepatic and peripheral energy homeostasis.

### Deletion of *Oct1* altered thiamine disposition and protected mice from beriberi

**Fig 3:**
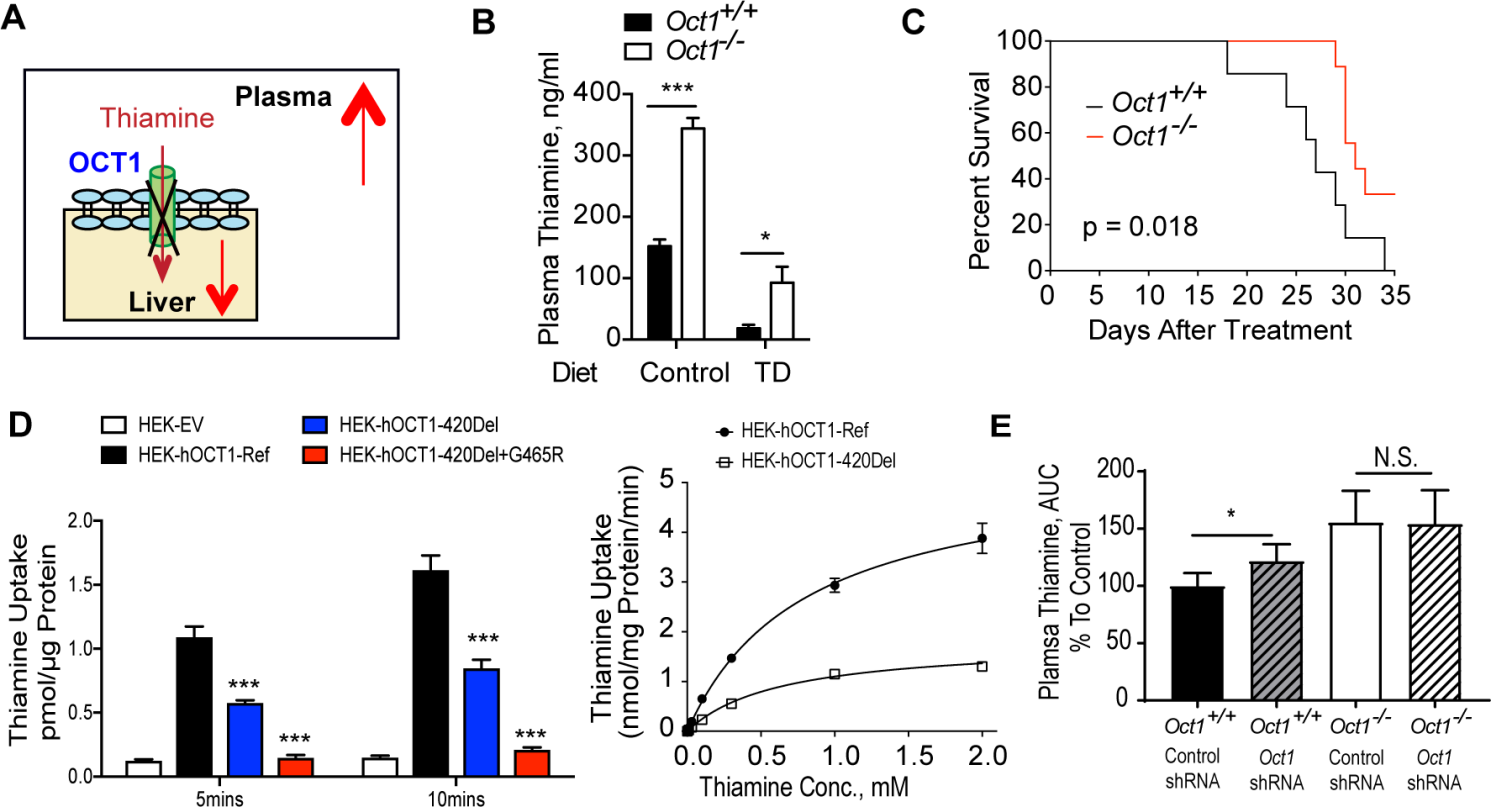
*Oct1* function modulated thiamine disposition in vivo and in vitro. (A) Scheme: deletion of *Oct1* reduces hepatic uptake of thiamine and increases plasma thiamine levels. (B) Plasma thiamine levels (*n* = 6 per genotype on a control diet; *n* = 7 per genotype on a TD). (C) Survival curves for mice on TDs (*n* = 7 for *Oct1*^*+/+*^ mice and *n* = 9 for *Oct1*^*-/-*^ mice). Animals were euthanized once the humane end points (body condition score of 2 or less or 15% body weight loss) were reached during the treatment (Gehan-Breslow-Wilcoxon test and log-rank test were used for analysis). (D) Representative graph of thiamine uptake in cells stably expressing EV, hOCT1-Ref, hOCT1-420Del, and hOCT1-420Del+G465R; a total of 25 nM thiamine was included in the uptake buffer. Representative graph of thiamine kinetics in cells expressing hOCT1-Ref and hOCT1-420Del; concentrations ranged from 25 nM to 2 mM; uptake was performed for 4 minutes. *n* = 3 replicated wells; two separate experiments were performed for the in vitro studies. (E) The area under the plasma concentration-time curve of thiamine. A single intraperitoneal injection of 2 mg/kg thiamine (with 4% ^3^H-thiamine) was administered to four groups of mice (*Oct1*^*+/+*^ mice treated with control shRNA, *n* = 6; *Oct1*^*+/+*^ mice treated with *Oct1* shRNA, *n* = 6; *Oct1*^*-/-*^ mice treated with control shRNA, *n* = 3 and *Oct1*^*-/-*^ mice treated with *Oct1* shRNA, *n* = 3) Data are normalized to *Oct1*^*+/+*^ mice treated with control shRNA. Data shown are mean ± SEM. Data were analyzed by unpaired two-tailed Student *t* test; *p < 0.05, **p < 0.01, and ***p < 0.001. Underlying data are provided in S1 Data. AUC, area under the curve; EV, empty vector; hOCT1-Ref, human OCT1 reference; hOCT1-420Del, human OCT1 with methinone_420_ deletion; hOCT1-420Del+G465R, human OCT1 with mutation in glycine_465_-to-arginine in addition to 420Del; *Oct1*, organic cation transporter 1; shRNA, short hairpin RNA; TD, thiamine deficient diet.

We hypothesized that the systemic plasma levels of thiamine are higher in *Oct1*^*-/-*^ mice as a result of reduced hepatic extraction of dietary thiamine (Fig 3A). As expected, *Oct1*^*-/-*^ mice had significantly higher plasma levels of thiamine (Fig 3B and S3A Fig) compared to *Oct1*^*+/+*^ mice on thiamine-controlled and thiamine-enriched diets. In addition, *Oct1* deletion preserved plasma thiamine levels in mice on TDs (Fig 3B). Thiamine deficiency is associated with life-threatening diseases, such as beriberi and Wernicke-Korsakoff syndrome [15, 41]. We hypothesized that preserved circulating thiamine levels would delay the development of severe thiamine deficiency syndromes and increase the rate of survival when mice were challenged with a TD. As shown in Fig 3C, there was a significant improvement in the overall survival of *Oct1*^*-/-*^ mice (*p* = 0.012, Gehan-Breslow-Wilcoxon test; *p* = 0.018, log-rank test) compared to *Oct1*^*+/+*^ mice. Modulation of *Oct1* expression levels provides a means of studying the effect of hepatic thiamine levels per se as opposed to systemic thiamine levels or thiamine levels in other tissues. Manipulation of dietary thiamine may have additional effects, for example, in the central nervous system. Notably, Liu and colleagues determined that reduced levels of thiamine in the systemic circulation in mice resulted in neurological effects in the hypothalamus, with anorexia and resultant reduction in peripheral adiposity [42].

In human populations, the *OCT1* gene is highly polymorphic [7–9, 43]. Many loss-of-function polymorphisms of *OCT1* have been characterized and found to affect hepatic uptake of drugs, leading to altered treatment response [43]. Here, in the uptake studies, cells expressing human *OCT1* genetic variants (420Del or 420Del+G465R) had significantly reduced uptake of thiamine compared to the reference allele (Fig 3D), although they have comparable levels of *OCT1* transcript (S3C Fig). In kinetic studies performed at 4 minutes, the maximum velocity (*V*_*max*_) of thiamine in cells expressing human OCT1 with methinone_420_ deletion (hOCT1-420Del) was 70% lower than in cells expressing the human OCT1 reference (hOCT1-Ref) (1.80 ± 0.09 nmol/mg protein/minute versus 5.36 ± 0.30 nmol/mg protein/minute) (Fig 3D). In contrast to humans, who express *OCT1* primarily in the liver, mice express *Oct1* in both the liver and the kidney; therefore, deletion of *Oct1* in the kidney could potentially affect systemic levels of thiamine in mice. To address this limitation of the *Oct1* knockout mice as a model for humans, we used hydrodynamic tail vein injection of mouse *Oct1* short hairpin RNA (shRNA) lentiviral particle (or empty vector shRNA lentiviral particle as control) to specifically knock down *Oct1* in the liver in both *Oct1*^*+/+*^ and *Oct1*^*-/-*^ mice. Following a single intraperitoneal injection of 2 mg/kg thiamine (with 4% ^3^H-thiamine), we observed that the area under the plasma concentration-time curve (AUC) of thiamine was significantly greater in wild-type mice treated with *Oct1* shRNA lentiviral particles compared to wild-type mice treated with vector control shRNA lentiviral particles (Fig 3E). Although not significant, similar trends were observed in the maximum concentration (C_max_) values (S3D Fig). Notably, the *Oct1* shRNA did not affect *Oct1* expression levels in the kidney (S3D Fig). Compared to wild-type mice with *Oct1* shRNA lentiviral particle knockdown, higher systemic levels of thiamine were observed in *Oct1*^*-/-*^ mice (Fig 3E), potentially reflecting an incomplete *Oct1* knockdown (50% liver *Oct1* expression reduction, S3D Fig) or an additive effect of renal *Oct1* deletion in *Oct1*^*-/-*^ mice. The data provide strong evidence that reduction of OCT1 expression in the liver alone can result in increased systemic thiamine exposure. Although the liver plays a role in pre-systemic thiamine metabolism, it should be noted that thiamine is metabolized in most tissues in the body; therefore, other tissues, such as the intestine, may contribute to pre-systemic metabolism of the vitamin. Collectively, alterations in OCT1 function through genetic polymorphisms affect thiamine uptake and disposition.

### Deletion of *Oct1* disrupted hepatic glucose metabolism

**Fig 4:**
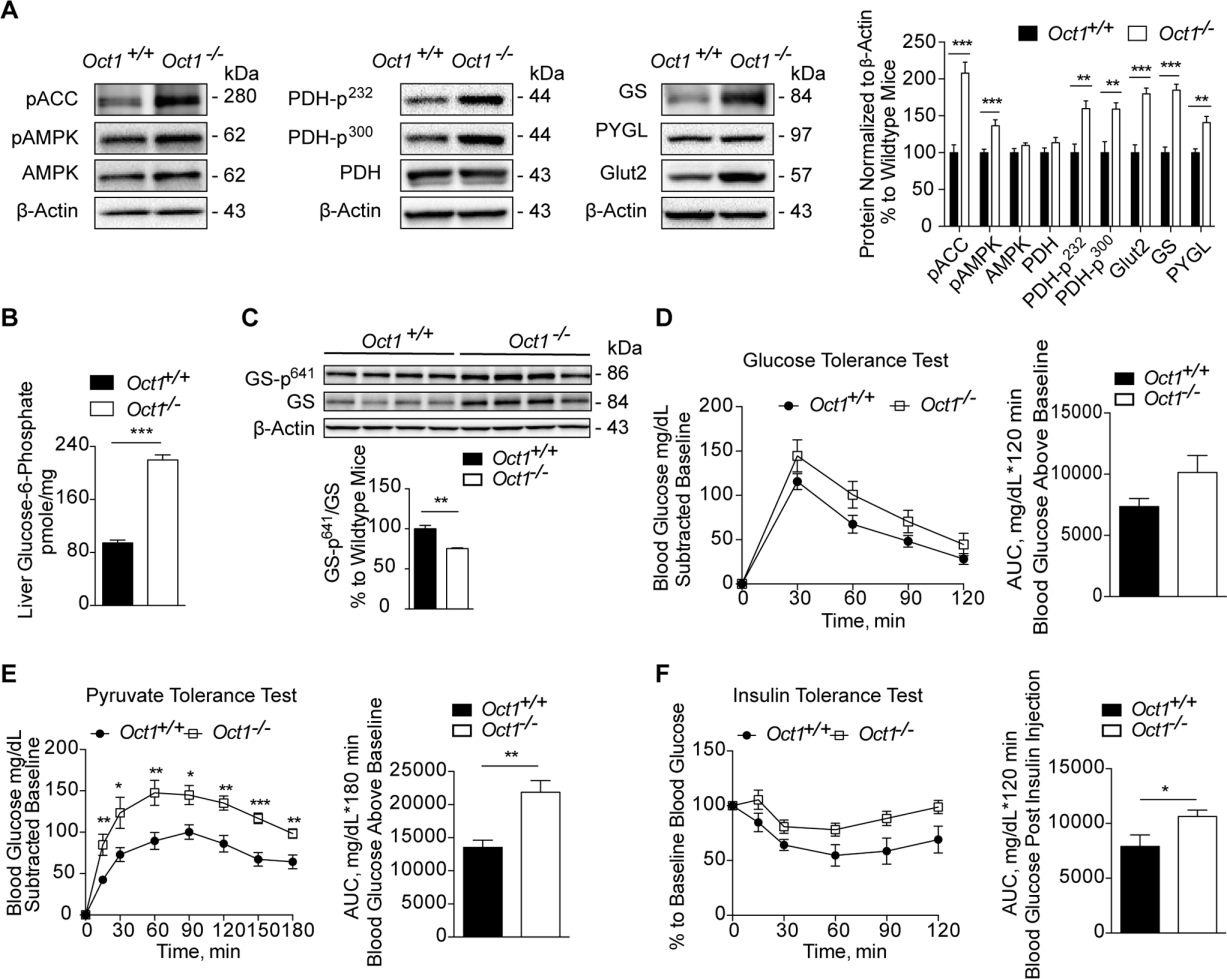
Deletion of *Oct1* altered hepatic glucose metabolism. (A) Representative western blots of key proteins involved in energy metabolism in mouse liver. Quantification of the western blots is shown in the right panel (*n* = 4–10 mice per genotype). (B) Hepatic glucose-6-phosphate levels (*n* = 4 per genotype). (C) Western blot of GS-p^641^, GS, and loading control β-actin. Mice were fasted overnight for 16 hours for (A), (B), and (C). (D) GTT in mice fasted for 5 hours, adjusted for baseline, and associated glucose AUC (*n* = 10 per genotype). (E) PTT in mice fasted for 16 hours, adjusted for baseline, and associated glucose AUC (*n* = 6 per genotype). (F) ITT in mice fasted for 5 hours and associated glucose AUC (*n* = 6 per genotype). Data shown are mean ± SEM. Data were analyzed by unpaired two-tailed Student *t* test; *p < 0.05, **p < 0.01, and ***p < 0.001. Underlying data are provided in S1 Data. AMPK, AMP-activated protein kinase; AUC, area under the curve; Glut2, glucose transporter 2; GTT, glucose tolerance test; GS, glycogen synthase; GS-p^641^, phospho-glycogen synthase at S641; ITT, insulin tolerance test; *Oct1*, organic cation transporter 1; pACC, phosphorylate acetyl-CoA carboxylase; pAMPK, phosphorylate 5' adenosine monophosphate-activated protein kinase; PDH, pyruvate dehydrogenase; PDH-p, phospho-pyruvate dehydrogenase; PTT, pyruvate tolerance test; PYGL; glycogen phosphorylase.

Our previous studies indicated that *Oct1* deletion resulted in reduced hepatic thiamine levels and levels of TPP [14], the cofactor of PDH. It is shown that reduced TPP levels directly affect the activity of PDH [44, 45]. As PDH plays a key role in energy metabolism linking glycolysis to the tricarboxylic acid (TCA) cycle and fatty acid metabolism [17], we hypothesized that the activity of hepatic PDH was impaired in *Oct1*^*-/-*^ mice. Because phosphorylation of PDH results in inactive forms of the enzyme [46], we measured levels of phosphorylated PDH (at two phosphorylation sites, Ser^232^ and Ser^300^) and mRNA levels of pyruvate dehydrogenase kinase 4 (PDK4). Both phosphorylated PDHs and *PDK4* transcripts were significantly higher in livers from *Oct1*^*-/-*^ mice (Fig 4A and S4A Fig). In addition, in *Oct1*^*-/-*^ mice, glycogen synthase (GS), and glucose transporter 2 (Glut2) were present at significantly higher levels (Fig 4A). Although glycogen phosphorylase (PYGL), which plays a key role in breakdown of hepatic glycogen, was also expressed at higher levels, the ratio of GS to PYGL was significantly higher in livers from *Oct1*^*-/-*^ mice (Fig 4A and S4B and S4C Fig). These data suggest that *Oct1*^*-/-*^ mice had higher rates of glycogen synthesis, which could explain the higher hepatic glycogen content in *Oct1*^*-/-*^ mice.

Our data suggested that livers from *Oct1*^*-/-*^ mice would have less activity of PDH, which in turn would result in a lower rate of conversion of pyruvate to acetyl-CoA entering the TCA cycle [47, 48] and thus an overall reduction in oxidative phosphorylation of glucose. We hypothesized that the reduction of oxidative phosphorylation of glucose would increase the accumulation of the intermediates of gluconeogenic substrates. These intermediates would lead to increased gluconeogenesis as glycolysis and gluconeogenesis are reciprocally regulated and highly depend on the availability of gluconeogenic substrates [1, 49]. The levels of glucose-6-phosphate (G6P), a strong allosteric activator of GS [50], were 2.3-fold (*p* < 0.0001) higher in the livers of *Oct1*^*-/-*^ mice (Fig 4B). In addition, the ratio of phosphorylated GS to total GS was significantly lower in *Oct1*^*-/-*^ mice (Fig 4C), consistent with a higher activity of GS in *Oct1*^*-/-*^ mice. To further investigate the role of OCT1 in hepatic glucose metabolism, we performed three standard tests related to glucose homeostasis [51]. In the glucose tolerance test (GTT), the blood glucose rose following oral glucose dosing and fell back to normal in both *Oct1*^*+/+*^ and *Oct1*^*-/-*^ mice (S4D Fig), although the *Oct1*^*-/-*^ mice had higher blood glucose levels at baseline. After adjusting for baseline, there was a trend toward higher blood glucose levels and an overall greater glucose AUC after a bolus dose of glucose in *Oct1*^*-/-*^ mice (Fig 4D). The GTT indicated that both *Oct1*^*+/+*^ and *Oct1*^*-/-*^ mice could produce insulin in response to rising glucose. In contrast, pyruvate tolerance tests (PTTs) were different between *Oct1*^*+/+*^ and *Oct1*^*-/-*^ mice (Fig 4E and S4E Fig). In particular, blood glucose was significantly higher at each time point after pyruvate injection in *Oct1*^*-/-*^ mice, which suggested that *Oct1*^*-/-*^ mice had higher rates of hepatic gluconeogenesis. In the insulin tolerance test (ITT), there was a trend toward higher blood glucose levels after insulin injection in the *Oct1*^*-/-*^ mice and an overall greater glucose AUC (Fig 4F). Blood glucose levels are maintained by glucose uptake mainly in peripheral tissues and glucose output primarily from the liver [52]. Data from the PTT suggested that the knockout mice had significantly higher hepatic gluconeogenesis, which may have contributed to the higher glucose exposure in *Oct1*^*-/-*^ mice following the ITT.

### Thiamine deficiency impaired glucose metabolism

**Fig 5:**
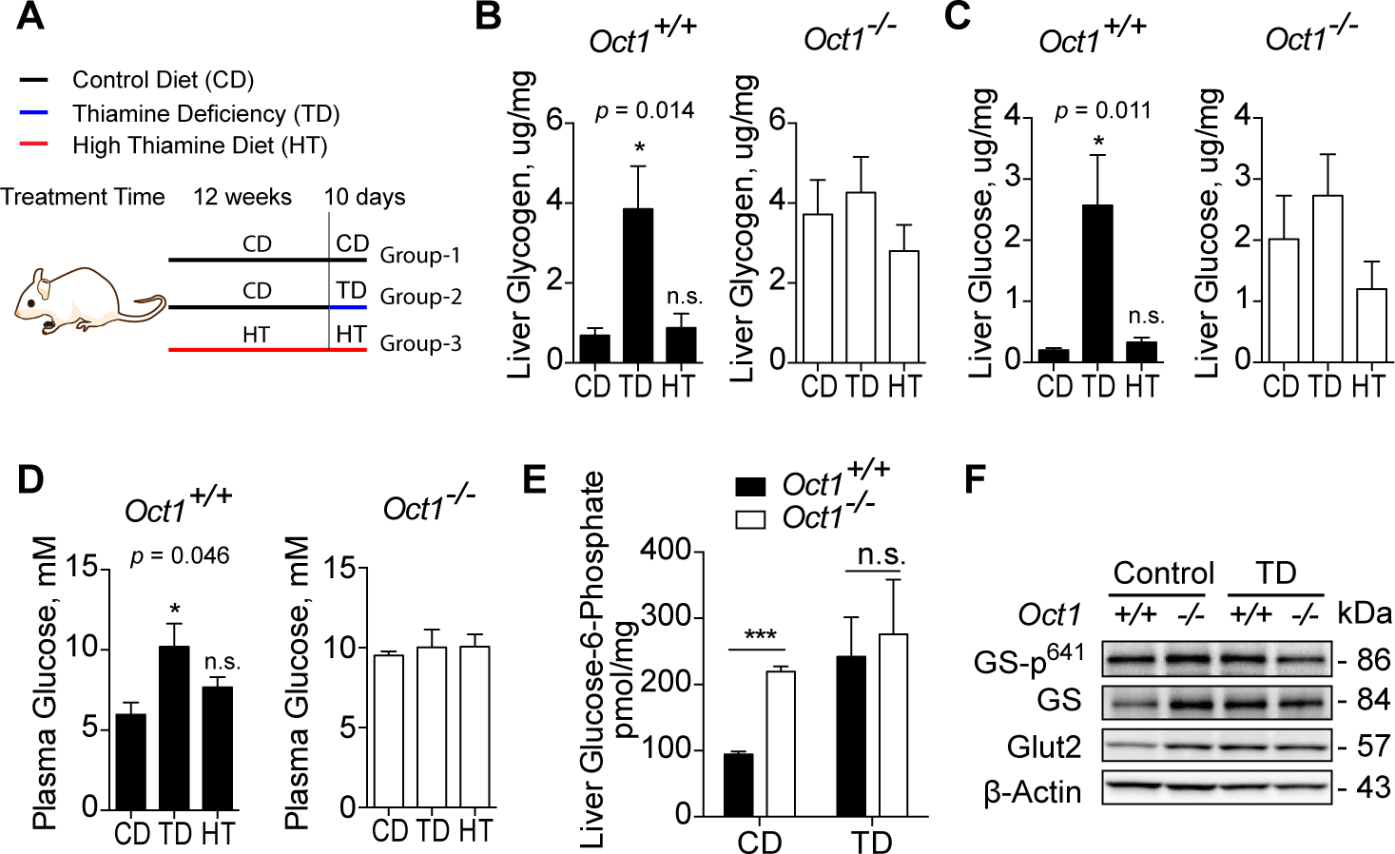
Different thiamine treatments affected glucose metabolism. (A) Scheme of experimental design. Two groups of mice (Group-1 and Group-2) were treated with a CD, 5 mg/kg, and one group (Group-3) with an HT, 50 mg/kg, to the end of the experiment. The third group of mice (Group-2) was treated with a CD but switched to a TD, 0 mg/kg, for 10 days. After dietary treatment, mice were fasted overnight for 16 hours before being humanely killed (*n* = 4 per genotype in each treatment). (B) Hepatic glycogen content quantification. (C) Hepatic glucose content quantification. (D) Plasma glucose quantification. For (B–D), CD, TD, and HT indicate diet received by the mice during the final 10 days of treatment. (E) Hepatic glucose-6-phosphate content quantification. (F) Representative western blots of protein expression in enzymes involved in energy metabolism; protein was pooled from 4 mice per genotype. Data shown are mean ± SEM. Data were analyzed by ordinary one-way ANOVA and *p*-value is stated, and Dunnett’s post hoc test was used to compare to the control (CD) group for (B), (C), and (D). Data were analyzed by unpaired two-tailed Student *t* test for (E); *p < 0.05, **p < 0.01, and ***p < 0.001. Underlying data are provided in S1 Data. CD, thiamine controlled diet; Glut2, glucose transporter 2; GS, glycogen synthase; GS-p^641^, phospho-glycogen synthase at serine 641; HT, high thiamine diet; Oct1, organic cation transporter 1; TD, thiamine deficient diet.

To understand the role of thiamine in regulating glucose metabolism, age-matched mice were placed on dietary chow containing three different doses of added thiamine, following the experimental design shown in Fig 5A. Wild-type mice fed a TD for 10 days had higher levels of hepatic glycogen, hepatic glucose, and plasma glucose compared to mice fed control diets (Fig 5B–5D). In contrast, varying thiamine content in the diet resulted in no significant differences in hepatic glycogen, hepatic glucose, or plasma glucose levels among *Oct1*^*-/-*^ mice (Fig 5B–5D). Furthermore, wild-type mice fed TDs had similar levels of hepatic glycogen, hepatic glucose, and plasma glucose as *Oct1*^*-/-*^ mice irrespective of the thiamine content in their diets, consistent with the idea that *Oct1* deficiency mimics thiamine deficiency in wild-type mice. Levels of G6P, an activator of GS, were significantly higher in livers from wild-type mice fed a TD diet and were comparable to liver levels of G6P in *Oct1*^*-/-*^ mice irrespective of thiamine content in the diet (Fig 5E). As shown by western blotting (Fig 5F), livers from *Oct1*^*-/-*^ mice in the control thiamine diet group and from both *Oct1*^*+/+*^ mice and *Oct1*^*-/-*^ mice in the TD group had higher GS and Glut2 protein levels compared to *Oct1*^*+/+*^ mice in the thiamine control group. Taken together, our data suggest that thiamine deficiency impairs glucose metabolism in wild-type mice and that *Oct1* deficiency phenocopies thiamine deficiency in wild-type mice.

### *Oct1*^*-/-*^ mice had higher adiposity and altered lipid metabolism

**Fig 6:**
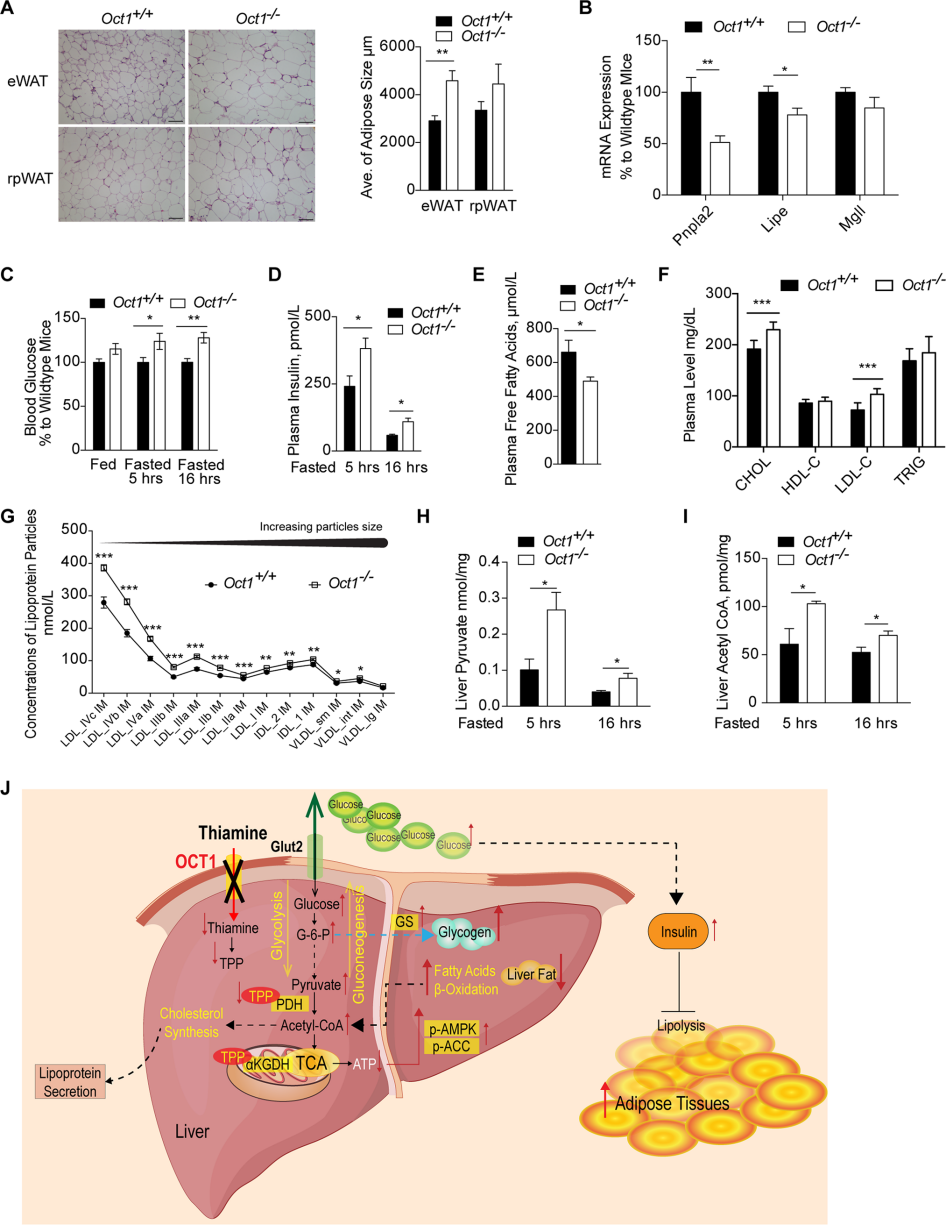
Deletion of *Oct1* modulated lipid metabolism. (A) H&E staining of adipose tissues and adipose cell size quantification (*n* = 3 per genotype). (B) mRNA expression of *Pnpla2*, *Lipe*, and *Mgll* in epididymal fat pads of mice fasted for 5 hours (*n* = 5 per genotype). (C) Blood glucose levels (*n* = 6 per genotype). (D) Plasma insulin levels (*n* = 9 or 10 per genotype in 5-hour fasted group; *n* = 4 per genotype in 16-hour fasted group). (E) Plasma free fatty acids levels in mice fasted for 5 hours (*n* = 9 or 10 per genotype). (F) Lipid panel showing plasma lipid levels in mice fasted for 5 hours (*n* = 9 or 10 per genotype). (G) Fractionation of the lipoprotein particles by size (*n* = 9 or 10 per genotype). (H) Hepatic pyruvate levels (*n* = 4 per genotype in 5-hour fasted group; *n* = 6 per genotype in 16-hour fasted group). (I) Hepatic acetyl-CoA levels (*n* = 4 per genotype in 5-hour fasted group; *n* = 6 per genotype in 16-hour fasted group). Data shown are mean ± SEM. Data were analyzed by unpaired two-tailed Student *t* test; *p < 0.05, **p < 0.01, and ***p < 0.001. Underlying data are provided in S1 Data. (J) Scheme of overall mechanism. The scheme illustrates how OCT1 deficiency affects disposition of thiamine and hence triggers a constellation of effects on hepatic and overall energy homeostasis. α-KGDH, α-ketoglutarate dehydrogenase; CHOL, cholesterol; CoA, coenzyme A; eWAT, epididimal white adipose tissue; Glut2, glucose transporter 2; HDL-C, High-density lipoprotein cholesterol; H&E, Haemotoxylin and Eosin; LDL-C, Low-density lipoprotein cholesterol; *Lipe*, lipase, hormone sensitive; *Mgll*, monoglyceride lipase; *Oct1*, organic cation transporter 1; p-ACC, phosphorylated acetyl co-A; p-AMPK, phosphorylated 5' adenosine monophosphate-activated protein kinase; PDH, pyruvate dehydrogenase; *Pnpla2*, patatin-like phospholipase domain-containing protein 2; rpWAT, retroperitoneal adipose tissue; TCA, tricarboxylic acid; TPP, thiamine pyrophosphate; TRIG, triglyceride.

*Oct1*^*-/-*^ mice exhibited increased adiposity (Fig 2C and 2D), and examination of fat cells through staining revealed significantly larger adipose cells in the epididymal fat pad (epididymal white adipose tissue [eWAT], *p* = 0.004) and a trend toward larger adipose cells in retroperitoneal adipose tissue (rpWAT) from *Oct1*^*-/-*^ mice (Fig 6A). To probe the mechanism of increasing adiposity and adipose cell size in the *Oct1*^*-/-*^ mice, we measured the mRNA expression levels of genes related to adipose metabolism. Fat gain may be due to imbalances between rates of TG synthesis and lipolysis. The mRNA expression of patatin-like phospholipase domain-containing protein 2 (*Pnpla2*) and lipase, hormone sensitive (*Lipe*) involved in adipose lipolysis was reduced in adipose tissue from *Oct1*^*-/-*^ mice compared to adipose tissue from *Oct1*^*+/+*^ mice (Fig 6B). In contrast, levels of genes involved in TG synthesis were similar between the two strains of mice (S5A Fig). *Pnpla2* (coding for adipose triglyceride lipase [ATGL]), *Lipe* (coding for hormone sensitive lipase [HSL]), and *Mgll* (coding for monoglyceride lipase [MGLL]) are responsible for three major steps in mobilizing fat through hydrolysis of TGs to release free fatty acids from the adipocytes [53]. Lower expression levels of these genes are consistent with lower rates of lipolysis in adipose tissue from *Oct1*^*-/-*^ mice. Insulin has antilipolytic effects in adipose tissue, regulating ATGL expression and promoting lipid synthesis, and chronic insulin treatment results in increased adipose mass [54, 55]. Corresponding to the higher levels of glucose (Fig 6C), we observed higher circulating levels of insulin in the *Oct1*^*-/-*^ mice (Fig 6D and S5B Fig), which suppressed lipolysis. Furthermore, fasting free fatty acid levels were lower in the plasma of *Oct1*^*-/-*^ mice (Fig 6E), which may reflect the lower rates of lipolysis in adipose tissue [56]. Data in *Oct1* knockout mice were corroborated by data from inbred strains of mice. In particular, *Oct1* mRNA levels in the liver inversely associated with percent fat growth and fat mass among various strains of mice (S1 Table). In addition, down-regulation of mitochondrial uncoupling protein 2 (*Ucp2*) was observed in brown adipose in *Oct1*^*-/-*^ mice (S5G Fig), which may associate with the reduced energy expenditure.

Examination of total cholesterol, HDL cholesterol, LDL cholesterol, and TG in plasma samples revealed significant differences in the two strains of mice. Notably, *Oct1*^*-/-*^ mice had higher plasma levels of total cholesterol and LDL cholesterol compared to *Oct1*^*+/+*^ mice, without significant differences in TG and HDL cholesterol (Fig 6F). The increase in LDL was due primarily to smaller LDL particles (Fig 6G). We observed no differences in the transcript levels of lipoprotein lipase (*Lpl*) and *Ldlr* in livers from *Oct1*^*+/+*^ mice and *Oct1*^*-/-*^ mice. However, livers from *Oct1*^*-/-*^ mice had higher transcript levels of 3-hydroxy-3-methylglutaryl-CoA reductase (*Hmgcr*), and Acyl-CoA: cholesterol acyltranferase 2 (*Acat2*) (S5E Fig). Consistent with lower activity of PDH, pyruvate levels were significantly higher in the livers from *Oct1*^*-/-*^ mice, as less pyruvate was converted to acetyl-CoA. Interestingly, contrary to our expectation, *Oct1*^*-/-*^ mice had higher levels of acetyl-CoA in their livers (Fig 6H and 6I). The higher accumulated acetyl-CoA may have resulted from higher fatty acid β-oxidation in *Oct1*^*-/-*^ mice [17, 48]. Our data suggest that up-regulation of enzymes involved in cholesterol synthesis and higher levels of the substrate precursor, acetyl-CoA, in the liver of *Oct1*^*-/-*^ mice result in alterations in hepatic cholesterol metabolism, leading to increased production of LDL particles. Furthermore, lower thiamine levels were correlated with higher levels of cholesterol in plasma and liver in male mice from various inbred strains of mice (S2 Table and S6 Fig).

## Discussion

Through extensive characterization of *Oct1* knockout mice, our data provide compelling evidence that *Oct1* deficiency leads to a constellation of diverse effects on energy metabolism that are consistent with GWAS demonstrating strong associations between OCT1 polymorphisms and a variety of metabolic traits in humans (Fig 1 and Table 1). Our data support the notion that hepatic thiamine deficiency is the underlying mechanism for the phenotypes associated with reduced OCT1 function. Five major effects of *OCT1* deficiency emerge from the current study: (1) a shift in the pathway of energy production from glucose to fatty acid oxidation due to lower activity of key thiamine-dependent enzymes in the liver; (2) increased gluconeogenesis and hepatic glucose output, with associated increases in liver glycogen and glucose levels; (3) increased peripheral adiposity stemming from alterations in energy metabolism; (4) changes in hepatic cholesterol homeostasis and plasma lipids that may contribute to cardiovascular disease risk; and (5) beneficial effects on life-threatening thiamine deficiency syndromes.

As the major energy-generating organ, the liver has high metabolic flexibility in selecting different substrates to use in energy production in response to various metabolic conditions. The glucose–fatty acid cycle, first proposed by Randle in 1963, plays a key role in regulating metabolic fuel selection, and impairment in metabolic flexibility contributes to insulin resistance and metabolic syndrome [17, 18, 47, 57, 58]. Many studies have shown that there is a failure to shift from fatty acid to glucose oxidation during the transition from fasting to feeding in individuals with obesity or diabetes [48, 57]. We observed lower hepatic steatosis (Fig 2A), largely due to increases in fatty acid oxidation in the liver [1, 59, 60]. Importantly, the observation that the *Oct1*^*-/-*^ mice had significantly lower RERs during the dark cycle than their wild-type counterparts suggests that overall, *Oct1*^*-/-*^ mice have a greater reliance on energy production from fatty acids over glucose during feeding than wild-type mice [61, 62] (S2J Fig). PDH is the key enzyme switch for the glucose–fatty acid cycle [17, 63]. Thus, alterations in its activity by reduced levels of the cofactor TPP in *Oct1*^*-/-*^ mice (Fig 4A) disrupt the hepatic glucose–fatty acid cycle, resulting in an impairment of hepatic energy homeostasis. In livers from *Oct1* deficient mice, β-oxidation of fatty acids becomes a major source of energy production, leading to impaired homeostasis in both lipid and carbohydrate metabolism.

In both the current study and our previous study [14], we observed increased levels of phosphorylated 5′ adenosine monophosphate-activated protein kinase (AMPK)and its downstream target, acetyl-CoA carboxylase (ACC), in livers from *Oct1*^*-/-*^ mice compared to livers from wild-type mice, indicative of a lower hepatic energy status in the knockout mice. As a result, fatty acid β-oxidation was stimulated in the livers from *Oct1*^*-/-*^ mice. However, as was evident by lower hepatic ATP content, the increased rates of fatty acid oxidation were not sufficient to compensate for normal rates of ATP production. Reduced glucose oxidation as well as a reduction in flux through the TCA cycle due to reduced activity of α-KGDH, another TPP-associated enzyme, may have contributed to the lower ATP production. Consistent with a lower flux through the TCA cycle as well as increases in β-oxidation of fatty acids, we observed higher levels of acetyl-CoA in livers from *Oct1*^*-/-*^ mice. Studies have shown that acetyl-CoA allosterically inhibits PDH, which results in further inhibition of glucose utilization [48, 58, 63]. Thus, in the livers from *Oct1*^*-/-*^ mice, this loop continued to stimulate fatty acid β-oxidation, which further suppressed hepatic glucose utilization, shifting the major energy source from glucose to fatty acids.

Increases in hepatic glycogen and glucose content in the *Oct1*^*-/-*^ mice (Fig 2A, Fig 5B and 5C) were likely due to changes in intermediary metabolites resulting directly from alterations in the activity of the TPP-dependent enzyme, PDH (Fig 4A). Reduced PDH activity resulted in higher levels of pyruvate in the liver of *Oct1*^*-/-*^ mice (Fig 6H), which is consistent with results from previous studies [63, 64]. Higher pyruvate levels can drive hepatic gluconeogenesis, resulting in increased hepatic glucose production and associated increases in hepatic glucose and glycogen levels [1, 52]. Whereas our data suggested that the glycogen accumulation in the livers of *Oct1*^*-/-*^ mice resulted from changes in key intermediate metabolites that are involved in hepatic energy metabolism, other regulatory paths such as hormonal, transcriptional, and neural regulation need to be further studied.

*Oct1*^*-/-*^ mice exhibited increased adiposity (Fig 2), which was more likely due to downstream effects of reduced transporter expression in the liver rather than in extra-hepatic tissues. Multiple factors contributed to the increased adiposity, such as hyperinsulinemia, hyperglycemia, increased hepatic glycogen, and reduced energy expenditure. Consistent with the increased adiposity observed in the *Oct1*^*-/-*^ mice, hepatic expression levels of *Oct1* are inversely correlated with fat growth and fat mass in inbred strains of mice (S1 Table). In addition, hyperinsulinemia in *Oct1* knockout mice may further result in increasing storage of TGs and suppression of lipolysis in peripheral adipose tissue, which reduced flux of fatty acids to the liver. Chronic insulin treatment has been shown to result in increased adipose mass due to suppression of lipolysis and increased lipid storage [54, 55], and in the current study, a high correlation between plasma insulin levels and fat mass in both wild-type and *Oct1*^*-/-*^ mice (S5D Fig) was observed. Furthermore, high insulin levels have been associated with low expression levels of the lipolytic enzyme *Pnpla2* in adipose tissue [55], consistent with results in the *Oct1*^*-/-*^ mice (Fig 6B), which is in agreement with the increased adiposity in these mice. In addition, high hepatic glycogen levels may have contributed to the increased adiposity. In particular, hepatic glycogen levels regulate the activation of the liver–brain–adipose axis [65]. Glycogen shortage during fasting triggers liver–brain–adipose neurocircuitry that results in stimulation of fat utilization. In contrast, in mice with elevated liver glycogen resulting from overexpression of GS or knockdown of PYGL, the liver–brain–adipose axis action is turned off, which preserves fat mass [65]. The greater stores of glycogen in the livers of *Oct1*^*-/-*^ mice may have shut off the liver–brain–adipose axis, contributing to the increased peripheral adiposity in the mice. Overall, our data in *Oct1*^*-/-*^ mice suggest that OCT1 plays a key role in regulation of peripheral metabolism, likely because of its effects on circulating glucose and insulin as well as increased stores of hepatic glycogen triggering a feedback loop mechanism between the liver, the brain, and the adipose tissue.

Parallels between phenotypes observed in GWAS in humans and those in the *Oct1*^*-/-*^ mice were striking. High plasma LDL, total cholesterol, and TG levels were observed in individuals with reduced-function polymorphisms of *OCT1* (R61C, F160L, G401S, V408M, 420del, and G465R) (Table 1) as well as in the *Oct1*^*-/-*^ mice (Fig 6F and 6G). In particular, the *Oct1*^*-/-*^ mice, and humans with reduced-function polymorphisms of OCT1 (S3 Table) [37], have increased levels of small dense LDL particles (Fig 6G), which in humans are predictive of increased risk of cardiovascular disease [66] and are a characteristic feature of the dyslipidemia associated with excess adiposity [67] and insulin resistance [68]. We speculate that the increased LDL levels in *Oct1*^*-/-*^ mice may result from a relative deficiency of hepatic thiamine. Specifically, livers from rats with thiamine deficiency have been shown to have lower TG but higher cholesterol content [69], consistent with our data in inbred strains of mice, in which inverse correlations were observed between plasma thiamine and cholesterol levels in both plasma and liver (S2 Table and S6 Fig). Importantly, our in vivo studies in mice showed that reduction of liver *Slc22a1* expression levels resulted in higher systemic thiamine levels (Fig 3B and 3E). Furthermore, individual OCT1 polymorphisms were nominally associated with systemic plasma levels of thiamine in humans (S4 Table) [70] as well as when combining six of the OCT1 nonsynonymous variants that were genotyped in the cohort by Rhee and colleagues (see S5 Table) [71]. Published studies in animals and humans suggest that thiamine supplementation may improve blood lipid profiles [24, 25]. Higher levels of the precursor for cholesterol synthesis, acetyl-CoA, as well as higher expression levels of enzymes involved in cholesterol synthesis [59, 72] (S5E Fig), potentially leading to greater rates of hepatic cholesterol production, could have contributed to the higher cholesterol and LDL levels in plasma. Although we were unable to detect differences in total hepatic cholesterol content between *Oct1*^*+/+*^ mice and *Oct1*^*-/-*^ mice (S5F Fig) corresponding to the observed differences in plasma cholesterol levels between the mouse strains, many factors that can modulate hepatic cholesterol content [73, 74], including perhaps increased export, need further investigation. Further studies are warranted to investigate the mechanisms underlying the effects of reduced OCT1 function as well as thiamine bioavailability on cholesterol and lipoprotein metabolism.

In addition to the metabolic changes that were observed in *Oct1*^*-/-*^ mice, the knockout mice were found to survive substantially longer on TDs. This may have been due to the higher systemic levels of thiamine (Fig 3B), which would spare essential organs such as the brain and heart from thiamine deficiency, as well as to the increased adiposity in the knockout mice (Fig 2C), which could protect the mice from the starvation that ensues from thiamine deficiency [42, 75, 76]. Nevertheless, the results have implications for human ancestors who harbored reduced-function genetic polymorphisms of OCT1. Because of differences in the tissue distribution of OCT1 between humans and mice, our study has limitations in directly extrapolating all the results obtained in mice to humans. In particular, because *Oct1* is also abundantly expressed in the kidney of mice, OCT1-mediated renal secretion of thiamine is another important determinant of systemic thiamine levels in mice. In contrast, in humans, OCT1 plays a role in modulating thiamine disposition largely in the liver and not in the kidney. Deletion of *Oct1*, particularly in the kidney of the knockout mice, therefore, may have modulated systemic thiamine levels and, thus, survival during TDs as well as other phenotypes observed in the current study. Today, thiamine deficiency is associated with aging, diabetes, alcoholism, and poor nutritional status [23, 77–79]. In the setting of thiamine deficiency, OCT1 reduced-function polymorphisms today would have mixed effects. On the one hand, individuals who harbored reduced-function variants would have higher systemic levels of thiamine, which may protect essential organs from thiamine depletion. On the other hand, the individuals would have low hepatic thiamine levels, which may predispose them to the deleterious effects of dysregulated plasma lipids and to obesity and diabetes (Table 1). In fact, lower thiamine levels have been reported in individuals with diabetes [23, 78] and, as noted, some studies have shown that high-dose thiamine supplementation has beneficial effects on diabetes [25, 80, 81].

Overall, the current study shows that OCT1 deficiency triggers a constellation of effects on hepatic and overall energy homeostasis (Scheme in Fig 6J). That is, reduced OCT1-mediated thiamine uptake in the liver leads to reduced levels of TPP and a decreased activity of key TPP-dependent enzymes, notably PDH and α-KGDH. As a result, there is a shift from glucose to fatty acid oxidation, which leads to imbalances in key metabolic intermediates, notably, elevated levels of pyruvate, G6P, and acetyl-CoA. Because of these imbalances, metabolic flux pathways are altered, leading to increased gluconeogenesis and glycogen synthesis in the liver. In addition, the increased acetyl-CoA levels along with elevated expression levels of key enzymes involved in cholesterol synthesis likely contribute to increases in plasma levels of total and LDL cholesterol observed in mice with *Oct1* deficiency and in humans with reduced-function genetic polymorphisms of OCT1. Although many of the details of the mechanisms have still to be worked out, our study provides critical insights into the role of thiamine in the liver in maintaining metabolic balance among energy metabolism pathways. Finally, our studies provide mechanistic insights into findings from GWAS implicating reduced-function variants in the *SLC22A1* locus as risk factors for lipid disorders and diabetes.

## Materials and methods

### Ethics statement

Animal experiments were approved by the Institutional Animal Care and Use Committee (IACUC) of University of California, San Francisco (AN119364), in accordance with the requirements of the National Research Council Guide for the Care and Use of Laboratory Animals and the Public Health Service Policy on the Humane Care and Use of Laboratory Animals. Humane end points were determined by body condition score of 2 or less or 15% body weight loss. Animals were euthanized once the humane end points were reached during the treatment in accordance with IACUC approved protocol. To limit pain and stress, mice were anesthetized deeply by isoflurane vaporizer and intraperitoneal injection of ketamine/medetomidine cocktail (75/1 mg/kg) prior to the physical cervical dislocation of euthanasia.

### Mining genetic association studies to identify phenotypes associated with SLC22A1 reduced-function variants

Various publicly available databases were used to determine whether there are significant genetic associations of *SLC22A1* reduced-function variants with human diseases and traits. The following databases were used: GWAS Catalog, dbGAP Association Results Browser, GRASP: Genome-Wide Repository of Associations Between SNPs [82], GIANT Consortium, Type 2 Diabetes Knowledge Portal, and Phenotypes and Genome Wide Associations Studies for Lipid Genetics. The first three databases, GWAS Catalog, dbGAP Association Results Browser, and GRASP, provide an easy to use interface to allow first-step information gathering of the types of human diseases and traits that have been reported in all published GWAS. Based on the results from the three databases, other specific databases relevant to the findings were then used. This includes searching for specific databases that have the GWAS summary statistics (beta coefficient and *p*-values). These are GIANT Consortium for body weight, Type 2 Diabetes Knowledge Portal for glucose and insulin traits, and all GWAS for lipid traits. These databases allow investigators to download the association studies for obtaining the *p*-values and the beta coefficients for the associations. In this study, we focused our search on nonsynonymous variants of OCT1 (SLC22A1) with minor allele frequencies ≥1% in populations with European ancestries (1000 Genome Project): R61C (rs12208357), F160L (rs683369), P341L (rs2282143), G401S (rs34130495), M408V (rs628031), 420Del (rs202220802) (rs662138 and rs1564348, which are in linkage disequilibrium to 420Del with r^2^ ≥ 0.77, D′ > 0.95), and G465R (rs34059508).

### Animal studies

All experiments on mice were approved by the IACUC of UCSF. *Oct1*^*-/-*^ mice were generated as previously described [83] and backcrossed more than 10 generations to FVB/N background. Mice were housed in a pathogen-free facility with a 12-hour light and 12-hour dark cycle and given free access to food and water. Five- or six-week-old experimental mice were fed with thiamine control diet Cat# TD.09549 (thiamine 5 mg/kg) containing 17.5% protein, 65.8% carbohydrate, and 5.0% fat by weight (Envigo, Madison, WI). Other thiamine diets contained the same composition as the thiamine control diet but differed in thiamine levels (TD Cat# TD.81029, 0 mg/kg; adjusted thiamine diet with different thiamine doses added, Cat# TD.120472, 25 mg/kg; Cat# TD.140164, 50 mg/kg). The periods of dietary treatments and time for mouse being humanely killed are indicated in the Results section and figure legends. The animal studies were conducted in male mice; however, overall body weight and liver weight were assessed in female mice. Mice treated with TD developed thiamine deficiency syndromes, resulting in reduction of food intake and body weight loss. During the treatment period, mice were closely monitored and weighed daily. Animals were euthanized once the humane end points (body condition score of 2 or less or 15% body weight loss) were reached during the treatment. To limit pain and stress, mice were anesthetized deeply by isoflurane vaporizer and intraperitoneal injection of ketamine/medetomidine cocktail (75/1 mg/kg) prior to the physical cervical dislocation of euthanasia.

### Hydrodynamic tail vein injection

Hydrodynamic tail vein injection procedure was conducted as described previously [84], with minor modifications. Briefly, the body weights of the mice were used to calculate the total volume (mL) required for injection based on the formula: body weight (g) * (mL/10g) + 0.1 mL (dead volume). The injection solution included 5*10^7 TU virus/mouse and saline to just the final volume. Instead of anesthetizing the mice, we used TransIT-QR kit MIR5210 (Mirus Bio LLC, US) and followed the online protocol (https://www.mirusbio.com/delivery/tailvein/). Dosing of ^3^H-thiamine was performed 48 hours after hydrodynamic tail vein injection. Mouse *Slc22A1* shRNA lentiviral particle (TRCN0000070156) and nonmammalian shRNA control pLKO.1 (SHC002V) were purchased from Sigma. Viruses were verified in HEK-293 cells. Briefly, pcDNA5 containing mouse *Slc22a1* was cotransfected with the *Slc22a1* knockdown vector or pLKO.1 control virus. mRNA was isolated after 48 hours transduction, and mRNA expression of Oct1 was measured.

### Body composition and metabolic cages

Before and during dietary treatment, body composition was determined by either quantitative magnetic resonance on the EchoMRI-3in1 body composition analyzer (EchoMRI, Houston, TX) or by DEXA. For DEXA, live animals were anesthetized with isoflurane and scanned on the Lunar PIXImus densitometer (Lunar PIXImus Corporation Headquarters, Madison, WI). After 8 weeks diet treatment, mice were placed in single housing cages for 3 days before initiating the CLAMS (Columbus Instruments, Columbus, OH) experiments. CLAMS was used to monitor food and water intake, oxygen consumption (VO_2_) and carbon dioxide production (VCO_2_), and locomotor activities for a period of 96 hours. All these experiments were performed in the Diabetes and Endocrinology Research Center Mouse Metabolism Core at UCSF.

### In vivo studies

Blood glucose levels from mice were measured using the FreeStyle Freedom Lite blood glucose meter (Abbott Laboratories, Chicago, IL) in samples obtained by the tail milking method. For oral glucose tolerance tests (OGTTs), mice were fasted for 5 hours and dosed with glucose 2 g/kg (Sigma-Aldrich, St. Louis, MO) by oral gavage. For ITTs, mice were fasted for 5 hours and dosed with 0.75 U/kg humulin R insulin 100 U/ml (Henry Schein Animal Health, Dublin, OH) by intraperitoneal injection. Blood was sampled at 0, 15, 30, 60, 90, and 120 minutes. For PTTs, mice were fasted overnight for 16 hours and dosed with pyruvate 2 g/kg (Sigma-Aldrich, St. Louis, MO) by intraperitoneal injection. Blood was sampled at 0, 15, 30, 60, 90, 120, 150, and 180 minutes.

### Tissue staining and histology

For adipose tissues and liver glycogen staining, mice were fasted for 16 hours and perfused with 20 mL 4% paraformaldehyde (PFA) in PBS. Epididymal fat pad, rpWATs, and liver were incubated in 4% PFA for 48 hours at 4°C and transferred to 70% ethanol. For Oil Red-O (ORO) staining in liver, mice were fasted for 16 hours and perfused with 10 mL PBS. Livers were fixed via sucrose infiltration steps prior to freezing. After incubating in 30% sucrose in PBS at 4°C for 24 hours, tissues were frozen in OCT molds. Fixed or frozen tissues were transferred to the histology and light microscopy core at Gladstone Institutes for staining, imaging, and analysis. For the cell-size analysis, hematoxylin and eosin-stained paraffin-embedded sections (https://labs.gladstone.org/histology/pages/section-staining-haematoxylin-and-eosin-staining) of mouse adipose tissues were imaged using a Nikon Eclipse E600 upright microscope equipped with a Retiga camera (QImaging, Vancouver, BC, Canada) and a Plan Fluor 20×/0.3NA objective. For each sample, four independent fields were imaged for analysis and adipocyte size was determined using ImageJ (v.2.0.0-rc-3) software (US National Institutes of Health) and the Tissue Cell Geometry macro (http://adm.irbbarcelona.org/image-j-fiji). For quantifying the lipid droplets, ORO stained frozen sections of mouse liver were imaged as above. For each sample, four independent fields were imaged for analysis. RGB images were then color thresholded to ORO, and the total area of ORO-positive pixels was summed for each image using the Analyze Particles function. For quantifying the glycogen levels in liver sections, Periodic-Acid Schiff’s stained mouse liver was imaged as above. For each sample, four independent fields were imaged and the mean intensity and integrated density values were averaged for each image using the Analyze Particles function.

### Metabolic parameters measurements

Mice were humanely killed and blood was collected via posterior vena cava to BD microtainer tubes with dipotassium EDTA (365974) or heparin (365985). Plasma was sent to the Clinical Laboratory of San Francisco General Hospital for measurement of total, LDL, and HDL cholesterol and TGs and liver panel. Plasma was send to Children's Hospital Oakland Research Institute for the measurement of lipoprotein particles size, as described previously [85]. Glucose (GAGO20), glycogen (MAK016), free fatty acid (MAK044), acetyl-Coenzyme A (MAK039), and G6P (MAK014) quantification kits were purchase from Sigma-Aldrich (St. Louis, MO). Pyruvate (ab65342), cholesterol (ab102515), and TG (ab65336) quantification kits were purchase from Abcam (Cambridge, MA). Plasma insulin was measure by ELISA (EMINS) from Thermo Fisher Scientific (Waltham, MA). Plasma was sent to Molecular MS Diagnostics, Inc. (Warwick, RI), for thiamine quantification, as previously described [14].

### Western blot analysis

Tissues were homogenized in CelLytic MT lysis buffer (Sigma-Aldrich, St. Louis, MO) with cOmplete ULTRA protease inhibitor tablet and PhosSTOP phosphatase inhibitor tablet freshly added (Roche). After normalization, equal protein amounts from each sample were loaded in to 4%–20% criterion Tris-HCl gel (Bio-Rad, Hercules, CA) and run at 110 V. Protein was transferred to EMD milllipore immobilon PVDF membranes at 100 V for 1 hour at 4°C using the criterion blotter. GS (15B1) (#3886; 1:1,000 dilution), phospho-glycogen synthase (Ser641) (#3891, 1:1,000 dilution), phospho-acetyl-CoA carboxylase (Ser79) (#3661; 1:2,000 dilution), AMPKα (23A3) (#2603, 1:1,000 dilution), and phospho-AMPKα (Thr172) (#2535; 1:1,500 dilution) were purchased from Cell Signaling Technology (Danvers, MA). Anti-PDH-E1α (pSer^232^) (#AP1063; 1:2,000 dilution) and Anti-PDH-E1α (pSer^300^) (#AP1064; 1:2,000 dilution) were purchased from EMD Millipore (Billerica, MA). PDH-E1α (D-6) (#sc-377092; 1:200 dilution) and β-actin (C4) (#sc-47778; 1:4,000 dilution) were purchased from Santa Cruz Biotechnology, Inc. (Dallas, TX). Anti-PYGL (#ab198268; 1:1,000 dilution) was purchased from Abcam (Cambridge, MA). Anti-Glut2 (#600-401-GN3; 1:1,000 dilution) was purchased from Rockland (Limerick, PA). Primary antibodies were incubated overnight at 4°C. Secondary antibodies, goat anti-rabbit IgG-HRP (#sc-2030; 1:5,000; Santa Cruz Biotechnology, Inc.), and anti-mouse IgG-HRP (#7076; 1:5,000; Cell Signaling Technology) were incubated for 2 hours at room temperature. Either Amersham ECL western blotting detection reagents (RPN2106) or ECL Prime western blotting detection reagents (RPN2232) were used for detection. Membranes were imaged by ProteinSimple western blot imaging system (San Jose, CA). For the quantification of western blot bands, the ImageJ (US National Institutes of Health) method was used.

### Gene expression analysis

Total RNA from mouse tissues or cell lines was isolated using RNeasy Mini kit (Qiagen, Valencia, CA). Total RNA (2 μg) from each sample was reverse transcribed into cDNA using SuperScript VILO cDNA Synthesis kit (Life Technologies, CA). Quantitative real-time PCR was carried out in 384-well reaction plates using 2X Taqman Fast Universal Master Mix (Applied Biosystems, Foster City, CA), 20X Taqman specific gene expression probes, and 10 ng of the cDNA template. The reactions were carried out on an Applied Biosystems 7900HT Fast Real-Time PCR System (Applied Biosystems, Foster City, CA). The relative expression level of each mRNA transcript was calculated by the comparative method (ΔΔCt method), normalized to the housekeeping gene, β-actin.

### Transporter uptake studies

The stably overexpressing pcDNA5 empty vector, mouse OCT1, human OCT1-reference, OCT1-420 del, and OCT1-420 del+G465R cell lines were maintained in Dulbecco’s Modified Eagle Medium (DMEM H-21) supplemented with hygromycin B (100 ug/mL) (Thermo Fisher Scientific, Waltham, MA), penicillin (100 U/mL), streptomycin (100 mg/mL), and 10% fetal bovine serum. Cell culture supplies were purchased from the Cell Culture Facility (UCSF, CA). Cells were cultured on poly-D-lysine coated 96-well plates for 24 hours to reach 95% confluence. Before the uptake experiments, the culture medium was removed and the cells were incubated in Hank’s balanced salt solution (HBSS) (Life Technology, CA) for 15 minutes at 37°C. Radiolabeled thiamine [3H(G)] hydrochloride (20 Ci/mmol) was purchased from American Radiolabeled Chemicals Incorporation (St. Louis, MO). Thiamine hydrochloride was purchased from Sigma-Aldrich (St. Louis, MO). Chemicals and radiolabeled compounds were diluted in the HBSS for uptake experiments. The details for drug concentrations and uptake time are described in the Results section and figure legends. The uptake was performed at 37°C, and then the cells were washed three times with ice-cold HBSS. After that, the cells were lysed with buffer containing 0.1 N NaOH and 0.1% SDS, and the radioactivity in the lysate was determined by liquid scintillation counting. For the transporter study, the Km and Vmax were calculated by fitting the data to a Michaelis-Menten equation using GraphPad Prism software 6.0 (La Jolla, CA).

### Statistical analysis

All mice were randomly assigned to the control or each treatment group. No statistical method was used to predetermine sample size, and sample size was determined on the basis of previous experiments. Numbers of mice for each experiment are indicated in figure legends. Mice that were dead or sick before the end of experiments were excluded from the final analysis. Investigators were not blinded during experiments. Data were expressed as mean ± SEM. Appropriate statistical analyses were applied, as specified in the figure legends. Data were analyzed using GraphPad Prism software 6.0 (La Jolla, CA). Differences were considered statistically significant at *p* < 0.05; *p < 0.05, **p < 0.01, and ***p < 0.001.

## Supporting information

S1 Fig Manhattan plots of the meta-analysis genome-wide association of SNPs. (A) Manhattan plots of the meta-analysis genome-wide association of SNPs with LDL cholesterol levels and (B) total cholesterol. The data are shown as −log10 *p*-value in up to 188,577 individuals with European Ancestry. The data are plotted using the results available from the Global Lipids Genetics Consortium, http://csg.sph.umich.edu/abecasis/public/lipids2013/. The names of the genes in the top locus for each chromosome were labeled. Over 100 loci were associated with lipids at *p* < 5 × 10^−8^, including *SLC22A1*, which is the top locus in chromosome 6. (C) The plot shows the correlation, R^2^, among the SNPs in the *SLC22A1*, *SLC22A2*, *SLC22A3*, *LPAL2*, and *LPA* genes. Darker red showed R^2^ > 0.8, whereas light color showed weak linkage. SNPs in SLC22A1 (on the top left region: rs12208357 [R61C], rs2282143 [P341L], rs683369, rs34130495 [G401S], rs628031 [V408M], rs662138 [LD to 420del], rs1443844, rs11753995, rs34059508 [G465R], rs2297374, rs1564348 [LD to 420del]) have very weak linkage, R^2^ < 0.1, with SNPs in LPAL2 and LPA genes region (bottom right region: rs3798220, rs7767084, rs10755578, rs6415084, rs74617384, rs3798221, rs191555775, rs55730499, rs41272114, rs10455872, rs7770628, rs9355814, rs7759633, rs1367211, rs1406888, rs2315065, rs186696265). One of the missense SNPs in OCT1, rs2282143 (P341L), has a weak correlation, r^2^ = 0.54, with a missense variant Ile1891Met (rs3798220). This plot was generated using LDLink, https://analysistools.nci.nih.gov/LDlink/?tab=ldmatrix. The R^2^ information is generated using genotype data from 1000 Genomes population from European Ancestry. G401S, Glycine to serine in amino acid position 401; G465R, Glycine to Arginine in amino acid position 465; I1891M, Isoleucine to methionine in amino acid position 1891; LD, linkage disequilibrium; LDL, low-density lipoprotein; *LPA*, lipoprotein(a); LPAL2, lipoprotein(a) like 2; OCT1, organic cation transporter 1; P341L, Proline to Leucine in amino acid position 341; R61C, Arginine to cysteine in amino acid position 61; *SLC*, solute carrier; V408M, Valine to methionine in amino acid position 408; 420del, methinone420 deletion.
(DOCX)

S2 Fig Altered energy metabolism in *Oct1*^*-/-*^ mice. (A) Lipid droplet quantification of ORO liver staining images (*n* = 3 per genotype). (B) Density quantification of PAS liver staining images for glycogen (*n* = 3 per genotype). (C) Quantified hepatic glucose for mice fasted 16 hours overnight (*n* = 10 per genotype). (D) Body weights for mice fasted 16 hours (*n* = 14 per genotype). (E) Percent of epididymal fat pad weight and liver weight to total body weight (*n* = 14 per genotype). Similar trends in liver weight, but not body weight, were observed in female mice (data not shown). (F) Respiratory O_2_ consumption normalized by lean body weight for 96 hours and calculated AUC. (G) Energy expenditure normalized by lean body weight for 96 hours and calculated AUC. (H) Activity counts for 96 hours, and associated summary of activity. (I) Food intake. (J) Respiratory exchange ratio, RER = VCO_2_/VO_2_ (*n* = 12 per genotype). (K) Liver function test (*n* = 5 per genotype). (L) mRNA expression levels for thiamine transporters in the liver (*n* = 6 per genotype). Data shown are mean ± SEM. Data were analyzed by unpaired two-tailed Student *t* test; *p < 0.05, **p < 0.01, and ***p < 0.001. Underlying data are provided in S1 Data. AUC, area under the curve; *Oct1*, organic cation transporter 1; ORO, Oil Red-O; O_2_, oxygen; PAS, Periodic Acid-Schiff; VCO_2_, carbon dioxide production; VO_2_, oxygen consumption.
(DOCX)

S3 Fig Effects of OCT1 and its genetic variants on thiamine disposition. (A) Plasma thiamine concentration (*n* = 6 per genotype in each diet). (B) Thiamine uptake in cells expressing EV and hOCT1-Ref. *n* = 3 replicated wells; two separate experiments performed for in vitro studies. (C) *Oct1* mRNA expression levels in cells stably expressing EV, hOCT1-Ref, hOCT1-420Del, and hOCT1-420Del+G465R (*n* = 3 wells per cell line). (D) Mouse Slc22A1 shRNA lentiviral particle knockdown experiments in mouse OCT1 overexpressing cells and wild-type mice. Data show mRNA levels in the livers and kidneys of control mice and mice that received a hydrodynamic tail vein injection of shRNA to OCT1. The maximal plasma concentration of thiamine. A single intraperitoneal injection of 2 mg/kg thiamine (with 4% ^3^H-thiamine) was administered to four groups of mice (*Oct1*^*+/+*^ mice treated with control shRNA, *n* = 6; *Oct1*^*+/+*^ mice treated with *Oct1* shRNA, *n* = 6; *Oct1*^*-/-*^ mice treated with control shRNA, *n* = 3; and *Oct1*^*-/-*^ mice treated with *Oct1* shRNA, *n* = 3) Data are normalized to *Oct1*^*+/+*^ mice treated with control shRNA. Data shown are mean ± SEM. Data were analyzed by unpaired two-tailed Student *t* test; *p < 0.05, **p < 0.01, and ***p < 0.001. Underlying data are provided in S1 Data. EV, empty vector; hOCT1-Ref, human OCT1 reference; hOCT1-420Del, human OCT1 with methinone_420_ deletion; hOCT1-420Del+G465R, human OCT1 with mutation in glycine_465_-to-arginine in addition to 420Del; OCT1, organic cation transporter 1; shRNA, short hairpin RNA; Slc, solute carrier.
(DOCX)

S4 Fig Deletion of *oct1* altered hepatic glucose metabolism. (A) *Pdk4* mRNA expression. (B) *Slc2a2* mRNA expression (*n* = 10 per genotype in mice fasted 16 hours; *n* = 6 per genotype in mice fasted 5 hours). (C) Ratio of GS to PYGL in protein expression (*n* = 8 mice per genotype). (D) GTT in mice fasted 5 hours (*n* = 10 per genotype). (E) PTT in mice fasted 16 hours (*n* = 6 per genotype). Data shown are mean ± SEM. Data were analyzed by unpaired two-tailed Student *t* test; *p < 0.05, **p < 0.01, and ***p < 0.001. Underlying data are provided in S1 Data. GS, glycogen synthase; GTT, glucose tolerance test; *oct1*, organic cation transporter 1; *Pdk4*, pyruvate dehydrogenase kinase 4; PTT, pyruvate tolerance test; PYGL, glycogen phosphorylase; *Slc*, solute carrier.
(DOCX)

S5 Fig Deletion of *Oct1* affected lipid metabolism. (A) mRNA expression of genes involved in energy metabolism in epididymal fat pads in mice fasted 5 hours (*n* = 5 per genotype). (B) Correlation between blood glucose and plasma insulin levels. (C) Correlation between plasma insulin and plasma free fatty acid levels. (D) Correlation between plasma insulin levels and epididymal fat pad mass. (E) mRNA expression of genes involved in cholesterol metabolism in mouse livers fasted 5 hours (*n* = 5 per genotype). (F) Hepatic total cholesterol content. (G) mRNA expression levels of genes involved in energy metabolism in brown adipose tissue. Data shown are mean ± SEM. Data were analyzed by unpaired two-tailed Student *t* test; *p < 0.05, **p < 0.01, and ***p < 0.001. Underlying data are provided in S1 Data. *Oct1*, organic cation transporter 1.
(DOCX)

S6 Fig Correlation of plasma thiamine levels with plasma lipid traits in inbred strains of mice. The data were obtained from previous studies conducted by the Aldon J. Lusis laboratory. (A) Correlation between plasma levels of thiamine and plasma LDL. (B) Correlation between plasma thiamine levels and plasma unesterified cholesterol. (C) Correlation between plasma thiamine levels and plasma esterified cholesterol. (D) Correlation between plasma thiamine levels and plasma total cholesterol. The figures were plotted using Pearson and Spearman correlation. Underlying data are provided in S1 Data. LDL, low-density lipoprotein.
(DOCX)

S1 Table Summary of association results for liver Slc22a1 mRNA expression with relevant traits among inbred strains of mice. Slc, solute carrier.
(DOCX)

S2 Table Summary of association results for plasma thiamine with relevant traits among inbred strains of mice. (DOCX)

S3 Table Association of OCT1 missense variants with circulating metabolites related to lipid particles. OCT1, organic cation transporter 1.
(XLSX)

S4 Table Associations of SLC22A1 reduced-function nonsynonymous variants (R61C, G401S, V408M, G465R) with thiamine levels in human. G401S, Glycine to serine in amino acid position 401; G465R, Glycine to arginine in amino acid position 465; R61C, Arginine to cysteine in amino acid position 61; SLC, solute carrier; V408M, Valine to methionine in amino acid position 408.
(XLSX)

S5 Table Associations of six SLC22A1 nonsynonymous variants (R61C, C88R, F160L, G401S, V408M, G465R) with thiamine levels in human. C88R, Cysteine to arginine in amino acid position 88; F160L, Phenylalanine to leucine in amino acid position 160; G401S, Glycine to serine in amino acid position 401; G465R, Glycine to Arginine in amino acid position 465; R61C, Arginine to cysteine in amino acid position 61; SLC, solute carrier; V408M, Valine to methionine in amino acid position 408.
(XLSX)

S1 Data Contains underlying data for figures. (XLSX)

## Abbreviations

*Acat2*: acyltranferase 2
ACC: acetyl-CoA carboxylase
α-KGDH: α-ketoglutarate dehydrogenase
AMPK: 5′ adenosine monophosphate-activated protein kinase
APOE: apolipoprotein E
ATGL: adipose triglyceride lipase
AUC: area under the curve
CLAMS: comprehensive laboratory animal monitoring system
C_max_: maximum concentration
dbGAP: database of Genotypes and Phenotypes
DEXA: dual-energy X-ray absorptiometry
DMEM: Dulbecco’s Modified Eagle Medium
eWAT: epididymal white adipose tissue
Glut2: glucose transporter 2
GRASP: Genome-Wide Repository of Associations Between SNPs and Phenotypes
GS: glycogen synthase
GTT: glucose tolerance test
GWAS: genome-wide association study
G6P: glucose-6-phosphate
HBSS: Hank’s balanced salt solution
*Hmgcr*: 3-hydroxy-3-methylglutaryl-CoA reductase
hOCT1-Ref: human OCT1 reference
hOCT1-420Del: human OCT1 with methinone_420_ deletion
HSL: hormone sensitive lipase
IACUC: Institutional Animal Care and Use Committee
ITT: insulin tolerance test
LDL: low-density lipoprotein
*Ldlr*: low-density lipoprotein receptor
*Lipe*: lipase, hormone sensitive
LPA: lipoprotein(a)
LPAL2: lipoprotein(a) like 2
*Lpl*: lipoprotein lipase
MGLL: monoglyceride lipase
OCT: organic cation transporter
OGTT: oral glucose tolerance test
ORO: Oil Red-O
O_2_: oxygen
PCSK9: proprotein convertase subtilisin/kexin type 9
PDH: pyruvate dehydrogenase
PDK4: pyruvate dehydrogenase kinase 4
PFA: paraformaldehyde
*Pnpla2*: patatin-like phospholipase domain-containing protein 2
PTT: pyruvate tolerance test
PYGL: glycogen phosphorylase
RER: respiratory exchange ratio
rpWAT: retroperitoneal adipose tissue
SLC22A1: solute carrier family 22 (organic cation transporter), member 1
shRNA: short hairpin RNA
TCA: tricarboxylic acid
TD: thiamine deficient diet
TG: triglyceride
TK: transketolase
TPP: thiamine pyrophosphate
*Ucp2*: uncoupling protein 2
VCO_2_: carbon dioxide production
*V_max_*: maximum velocity
VO_2_: oxygen consumption
420Del: OCT1 with methinone_420_ deletion
