# Supplementary figures and images for: Organic cation transporter 1 (OCT1) modulates multiple cardiometabolic traits through effects on hepatic thiamine content
Source: PLoS Biol. 2018 Apr 16;16(4):e2002907. doi: 10.1371/journal.pbio.2002907 (PMC5919692; doi:10.1371/journal.pbio.2002907)

**S1 Fig**

**
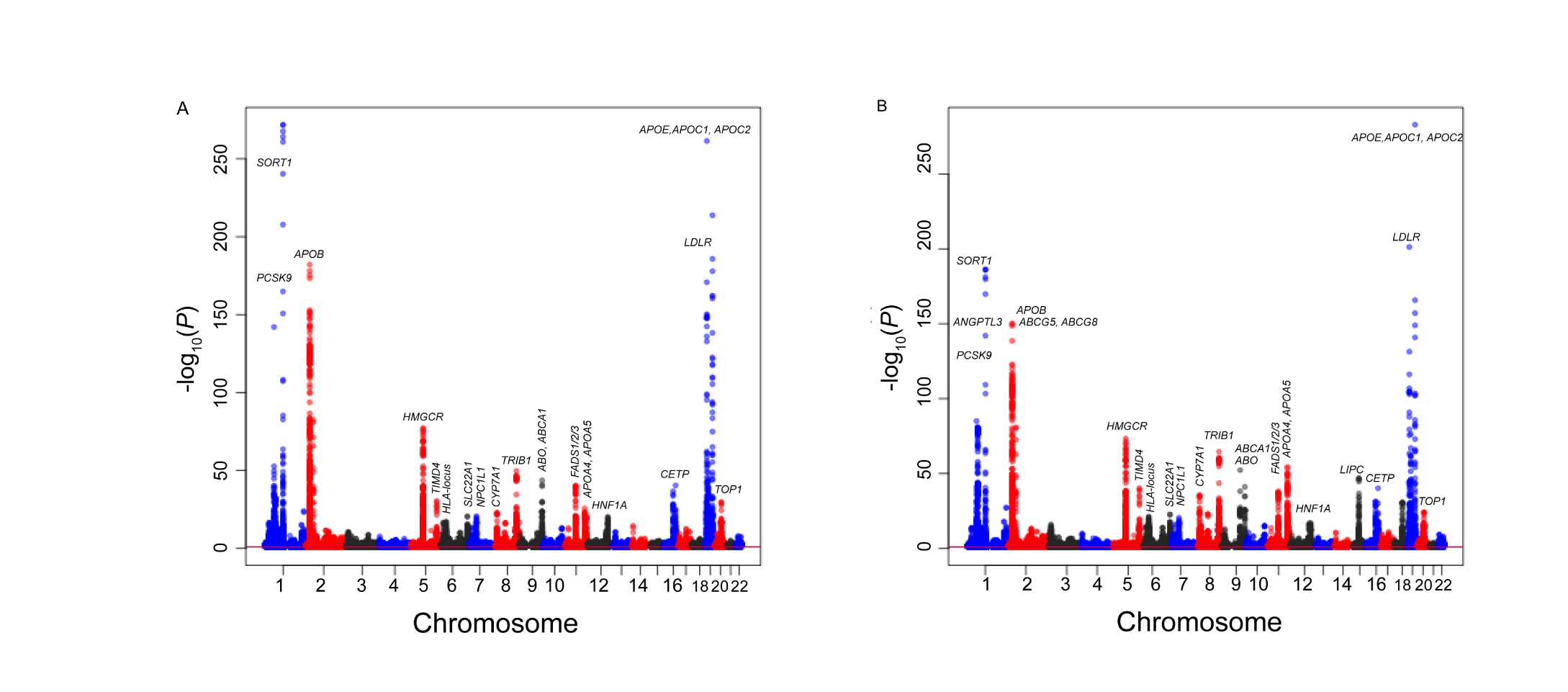
**

**
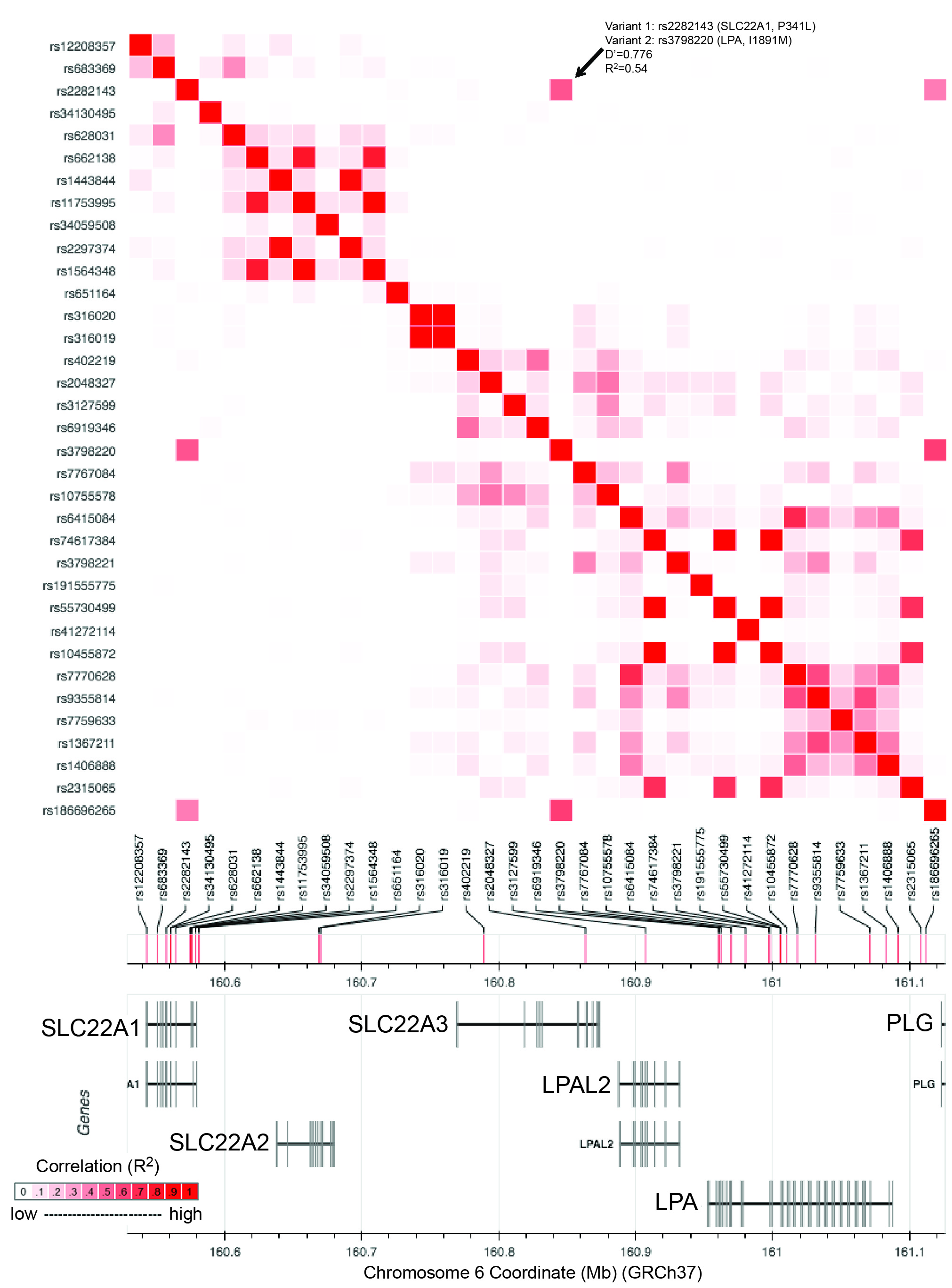
**

C

Supplement: S1 Fig — (A) Manhattan plots of the meta-analysis genome-wide association of SNPs with LDL cholesterol levels and (B) total cholesterol. The data are shown as −log10 p-value in up to 188,577 individuals with European Ancestry. The data are plotted using the results available from the Global Lipids Genetics Consortium, http://csg.sph.umich.edu/abecasis/public/lipids2013/. The names of the genes in the top locus for each chromosome were labeled. Over 100 loci were associated with lipids at p < 5 × 10^−8^, including SLC22A1, which is the top locus in chromosome 6. (C) The plot shows the correlation, R2, among the SNPs in the SLC22A1, SLC22A2, SLC22A3, LPAL2, and LPA genes. Darker red showed R2 > 0.8, whereas light color showed weak linkage. SNPs in SLC22A1 (on the top left region: rs12208357 [R61C], rs2282143 [P341L], rs683369, rs34130495 [G401S], rs628031 [V408M], rs662138 [LD to 420del], rs1443844, rs11753995, rs34059508 [G465R], rs2297374, rs1564348 [LD to 420del]) have very weak linkage, R2 < 0.1, with SNPs in LPAL2 and LPA genes region (bottom right region: rs3798220, rs7767084, rs10755578, rs6415084, rs74617384, rs3798221, rs191555775, rs55730499, rs41272114, rs10455872, rs7770628, rs9355814, rs7759633, rs1367211, rs1406888, rs2315065, rs186696265). One of the missense SNPs in OCT1, rs2282143 (P341L), has a weak correlation, r2 = 0.54, with a missense variant Ile1891Met (rs3798220). This plot was generated using LDLink, https://analysistools.nci.nih.gov/LDlink/?tab=ldmatrix. The R2 information is generated using genotype data from 1000 Genomes population from European Ancestry. G401S, Glycine to serine in amino acid position 401; G465R, Glycine to Arginine in amino acid position 465; I1891M, Isoleucine to methionine in amino acid position 1891; LD, linkage disequilibrium; LDL, low-density lipoprotein; LPA, lipoprotein(a); LPAL2, lipoprotein(a) like 2; OCT1, organic cation transporter 1; P341L, Proline to Leucine in amino acid position 341; R61C, Arginine to cysteine in amin [file pbio.2002907.s001.docx]

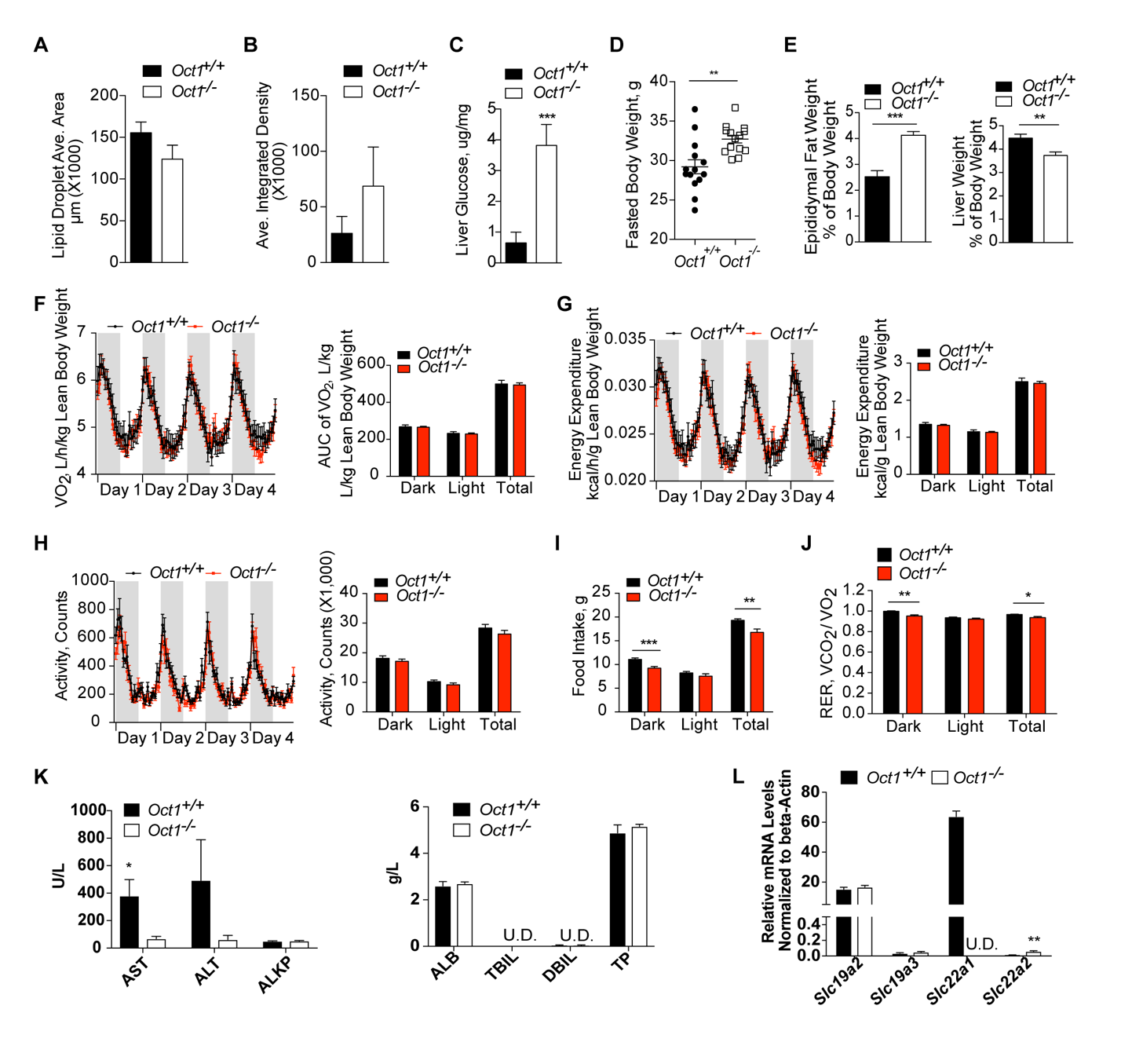

Supplement: S2 Fig — (A) Lipid droplet quantification of ORO liver staining images (n = 3 per genotype). (B) Density quantification of PAS liver staining images for glycogen (n = 3 per genotype). (C) Quantified hepatic glucose for mice fasted 16 hours overnight (n = 10 per genotype). (D) Body weights for mice fasted 16 hours (n = 14 per genotype). (E) Percent of epididymal fat pad weight and liver weight to total body weight (n = 14 per genotype). Similar trends in liver weight, but not body weight, were observed in female mice (data not shown). (F) Respiratory O2 consumption normalized by lean body weight for 96 hours and calculated AUC. (G) Energy expenditure normalized by lean body weight for 96 hours and calculated AUC. (H) Activity counts for 96 hours, and associated summary of activity. (I) Food intake. (J) Respiratory exchange ratio, RER = VCO2/VO2 (n = 12 per genotype). (K) Liver function test (n = 5 per genotype). (L) mRNA expression levels for thiamine transporters in the liver (n = 6 per genotype). Data shown are mean ± SEM. Data were analyzed by unpaired two-tailed Student t test; *p < 0.05, **p < 0.01, and ***p < 0.001. Underlying data are provided in S1 Data. AUC, area under the curve; Oct1, organic cation transporter 1; ORO, Oil Red-O; O2, oxygen; PAS, Periodic Acid-Schiff; VCO2, carbon dioxide production; VO2, oxygen consumption. (DOCX) [file pbio.2002907.s002.docx]

**
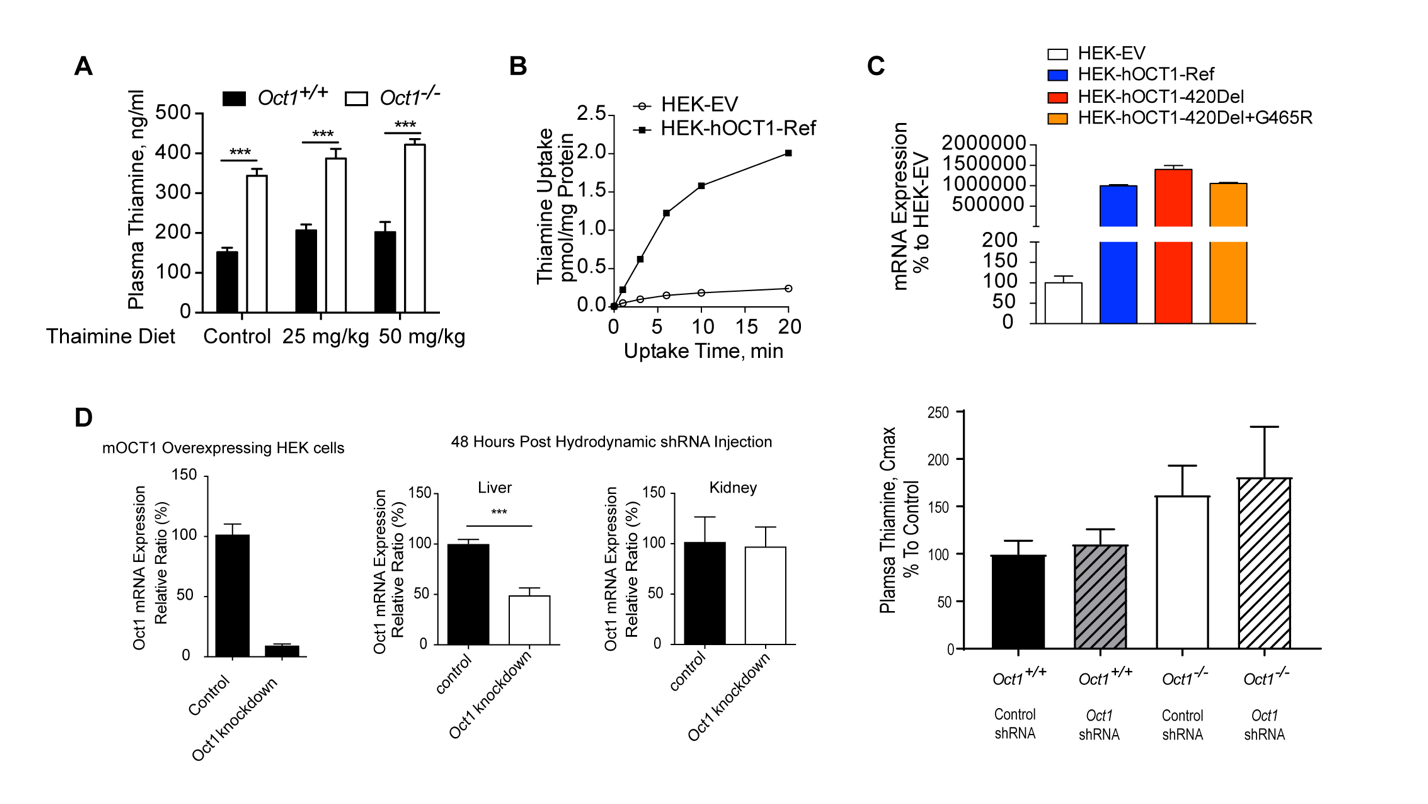
**

Supplement: S3 Fig — (A) Plasma thiamine concentration (n = 6 per genotype in each diet). (B) Thiamine uptake in cells expressing EV and hOCT1-Ref. n = 3 replicated wells; two separate experiments performed for in vitro studies. (C) Oct1 mRNA expression levels in cells stably expressing EV, hOCT1-Ref, hOCT1-420Del, and hOCT1-420Del+G465R (n = 3 wells per cell line). (D) Mouse Slc22A1 shRNA lentiviral particle knockdown experiments in mouse OCT1 overexpressing cells and wild-type mice. Data show mRNA levels in the livers and kidneys of control mice and mice that received a hydrodynamic tail vein injection of shRNA to OCT1. The maximal plasma concentration of thiamine. A single intraperitoneal injection of 2 mg/kg thiamine (with 4% 3H-thiamine) was administered to four groups of mice (Oct1+/+ mice treated with control shRNA, n = 6; Oct1+/+ mice treated with Oct1 shRNA, n = 6; Oct1-/- mice treated with control shRNA, n = 3; and Oct1-/- mice treated with Oct1 shRNA, n = 3) Data are normalized to Oct1+/+ mice treated with control shRNA. Data shown are mean ± SEM. Data were analyzed by unpaired two-tailed Student t test; *p < 0.05, **p < 0.01, and ***p < 0.001. Underlying data are provided in S1 Data. EV, empty vector; hOCT1-Ref, human OCT1 reference; hOCT1-420Del, human OCT1 with methinone420 deletion; hOCT1-420Del+G465R, human OCT1 with mutation in glycine465-to-arginine in addition to 420Del; OCT1, organic cation transporter 1; shRNA, short hairpin RNA; Slc, solute carrier. (DOCX) [file pbio.2002907.s003.docx]

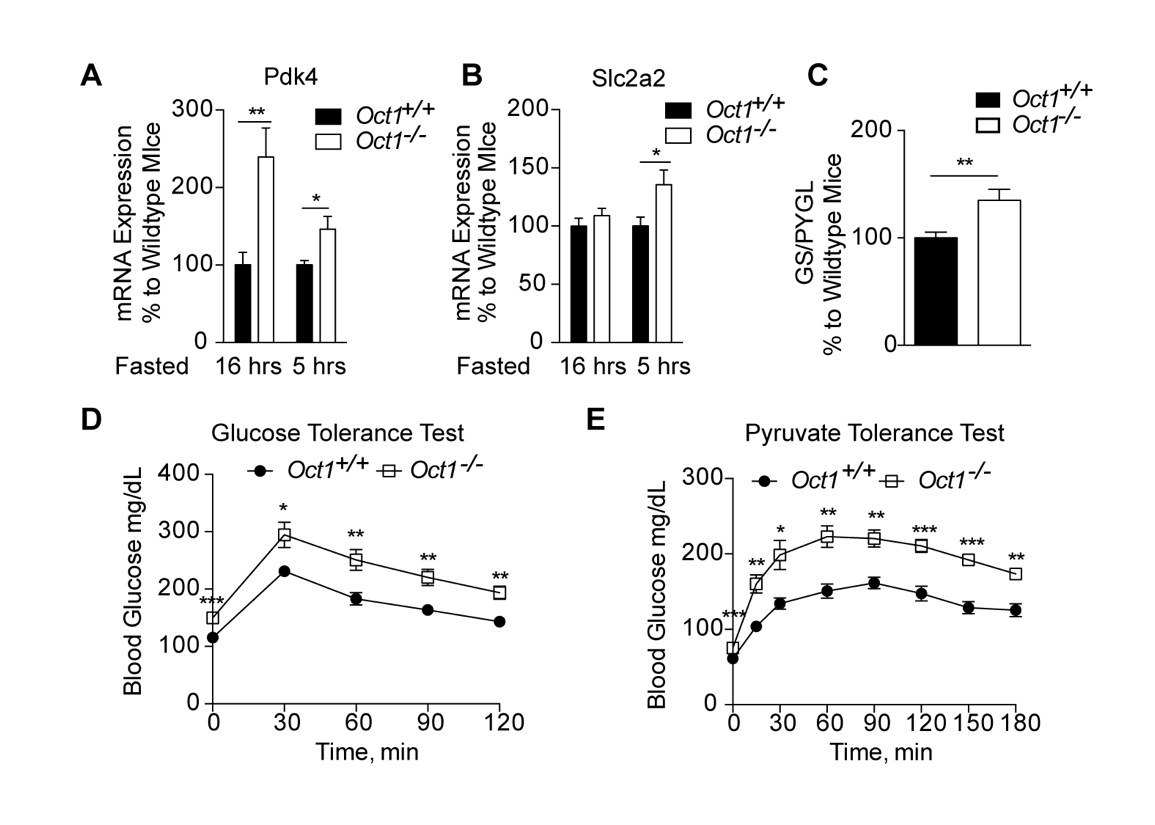

Supplement: S4 Fig — (A) Pdk4 mRNA expression. (B) Slc2a2 mRNA expression (n = 10 per genotype in mice fasted 16 hours; n = 6 per genotype in mice fasted 5 hours). (C) Ratio of GS to PYGL in protein expression (n = 8 mice per genotype). (D) GTT in mice fasted 5 hours (n = 10 per genotype). (E) PTT in mice fasted 16 hours (n = 6 per genotype). Data shown are mean ± SEM. Data were analyzed by unpaired two-tailed Student t test; *p < 0.05, **p < 0.01, and ***p < 0.001. Underlying data are provided in S1 Data. GS, glycogen synthase; GTT, glucose tolerance test; oct1, organic cation transporter 1; Pdk4, pyruvate dehydrogenase kinase 4; PTT, pyruvate tolerance test; PYGL, glycogen phosphorylase; Slc, solute carrier. (DOCX) [file pbio.2002907.s004.docx]

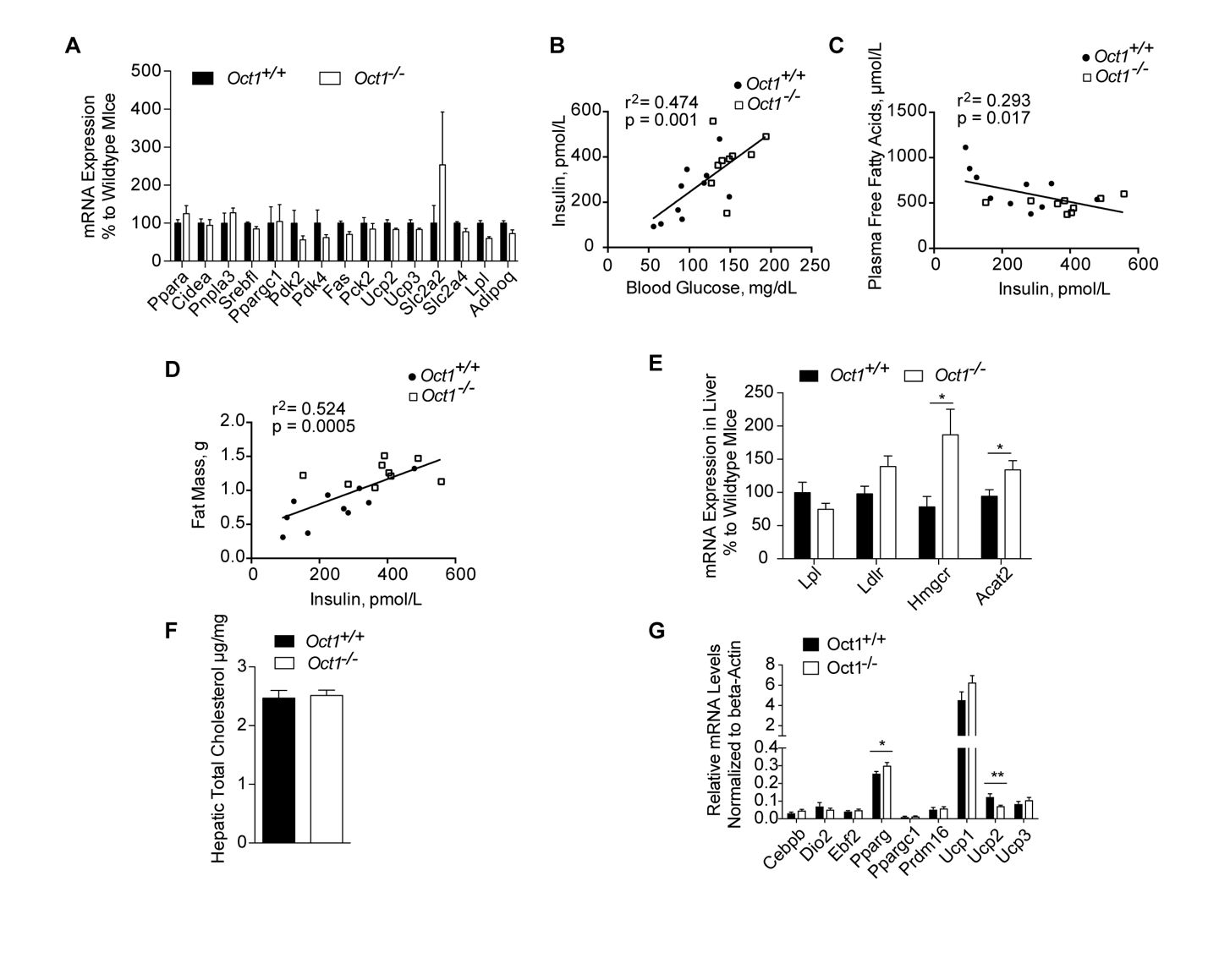

Supplement: S5 Fig — (A) mRNA expression of genes involved in energy metabolism in epididymal fat pads in mice fasted 5 hours (n = 5 per genotype). (B) Correlation between blood glucose and plasma insulin levels. (C) Correlation between plasma insulin and plasma free fatty acid levels. (D) Correlation between plasma insulin levels and epididymal fat pad mass. (E) mRNA expression of genes involved in cholesterol metabolism in mouse livers fasted 5 hours (n = 5 per genotype). (F) Hepatic total cholesterol content. (G) mRNA expression levels of genes involved in energy metabolism in brown adipose tissue. Data shown are mean ± SEM. Data were analyzed by unpaired two-tailed Student t test; *p < 0.05, **p < 0.01, and ***p < 0.001. Underlying data are provided in S1 Data. Oct1, organic cation transporter 1. (DOCX) [file pbio.2002907.s005.docx]

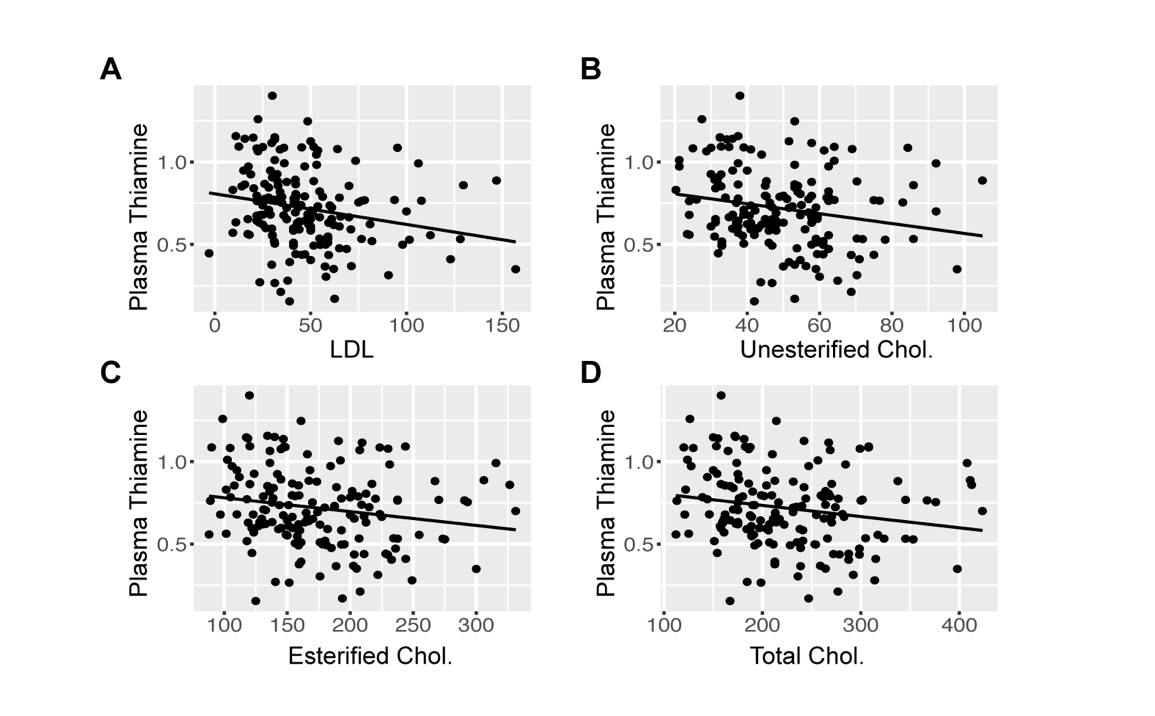

Supplement: S6 Fig — The data were obtained from previous studies conducted by the Aldon J. Lusis laboratory. (A) Correlation between plasma levels of thiamine and plasma LDL. (B) Correlation between plasma thiamine levels and plasma unesterified cholesterol. (C) Correlation between plasma thiamine levels and plasma esterified cholesterol. (D) Correlation between plasma thiamine levels and plasma total cholesterol. The figures were plotted using Pearson and Spearman correlation. Underlying data are provided in S1 Data. LDL, low-density lipoprotein. (DOCX) [file pbio.2002907.s006.docx]
