# Supplementary material for: Organic cation transporter 1 (OCT1) modulates multiple cardiometabolic traits through effects on hepatic thiamine content
Source: PLoS Biol. 2018 Apr 16;16(4):e2002907. doi: 10.1371/journal.pbio.2002907 (PMC5919692; doi:10.1371/journal.pbio.2002907)
Supplement: S1 Table — Slc, solute carrier. (DOCX) [file pbio.2002907.s007.docx]

**S1 Table: Summary of association results for liver Slc22a1 mRNA expression with relevant traits among inbred strains of mice.**

| **Mouse Sex** | **Tissue** | **Gene** | **Trait** | **bicor** | **p value** |
| --- | --- | --- | --- | --- | --- |
| Male | Liver | Slc22a1 | Liver Wt | 0.28710 | 0.00001 |
| Male | Liver | Slc22a1 | Liver/NMR_Mass_8wks | 0.22799 | 0.00055 |
| Male | Liver | Slc22a1 | Triglycerides (liver) | 0.28966 | 0.00195 |
| Male | Liver | Slc22a1 | Fat Mass_Percent Growth_2 to 4 wks | -0.20046 | 0.00361 |
| Male | Liver | Slc22a1 | Fat Mass_Percent Growth_2 to 8 wks | -0.24932 | 0.00022 |
| Male | Liver | Slc22a1 | Fat Mass_Percent Growth_2 to 6 wks | -0.21814 | 0.00163 |
| Male | Liver | Slc22a1 | Fat Mass_Percent Growth_4 to 8 wks | -0.20299 | 0.00243 |
| Male | Liver | Slc22a1 | Fat Mass_Percent Growth_6 to 8 wks | -0.26390 | 0.00008 |
| Male | Liver | Slc22a1 | BF_Percent Growth_0 to 4 wks | -0.20476 | 0.00249 |
| Male | Liver | Slc22a1 | BF_Percent Growth_0 to 6 wks | -0.21771 | 0.00128 |
| Male | Liver | Slc22a1 | BF_Percent Growth_0 to 8 wks | -0.23787 | 0.00033 |
| Male | Liver | Slc22a1 | BF_Percent Growth_2 to 4wks | -0.26685 | 0.00011 |
| Male | Liver | Slc22a1 | BF_Percent Growth_2 to 6wks | -0.27034 | 0.00008 |
| Male | Liver | Slc22a1 | BF_Percent Growth_2 to 8wks | -0.29944 | 0.00001 |
| Male | Liver | Slc22a1 | BF_Percent Growth_6 to 8 wks | -0.25616 | 0.00014 |
| Female | Liver | Slc22a1 | Gonadal Fat Wt | -0.21670 | 0.00176 |
| Female | Liver | Slc22a1 | Visceral Fat | -0.19663 | 0.00472 |
| Female | Liver | Slc22a1 | Visceral Fat/NMR_Mass_8wks | -0.19662 | 0.00472 |
| Female | Liver | Slc22a1 | Gonadal_Fat/NMR_Mass_8wks | -0.21762 | 0.00168 |

* The data were obtained from previous studies conducted by Aldon J. Lusis laboratory[1-3]. Correlations were calculated with the biweight midcorrelation (bicor), which is robust to outliers and was used in the studies performed by Aldons J. Lusis laboratory[1]. A positive bicor means that the higher expression level of Slc22a1 mRNA is associated with higher levels of the measured trait. A negative bicor means that the higher expression level of Slc22a1 mRNA is associated with lower levels of the measured trait.

**Supplemental references**

1. Hui, S.T., et al., *The genetic architecture of NAFLD among inbred strains of mice.* Elife, 2015. **4**: p. e05607.

2. Parks, B.W., et al., *Genetic control of obesity and gut microbiota composition in response to high-fat, high-sucrose diet in mice.* Cell Metab, 2013. **17**(1): p. 141-52.

3. Parks, B.W., et al., *Genetic architecture of insulin resistance in the mouse.* Cell Metab, 2015. **21**(2): p. 334-46.
