# Supplementary material for: Organic cation transporter 1 (OCT1) modulates multiple cardiometabolic traits through effects on hepatic thiamine content
Source: PLoS Biol. 2018 Apr 16;16(4):e2002907. doi: 10.1371/journal.pbio.2002907 (PMC5919692; doi:10.1371/journal.pbio.2002907)
Supplement: S2 Table — (DOCX) [file pbio.2002907.s008.docx]

**S2 Table: Summary of association results for plasma thiamine with relevant traits among inbred strains of mice.**

| **Mouse Sex** | **Metabolite Name** | **Trait Name** | **bicor** | **P-value** |
| --- | --- | --- | --- | --- |
| Male | Thiamine | LDL | -0.324612972 | 0.00150 |
| Male | Thiamine | Unesterified cholesterol | -0.274601974 | 0.00773 |
| Male | Thiamine | Total cholesterol | -0.247459141 | 0.01678 |
| Male | Thiamine | Esterified cholesterol | -0.246770177 | 0.01710 |
| Male | Thiamine | HDL | -0.207385229 | 0.04730 |
| Male | Thiamine | Liver total cholesterol | -0.204359937 | 0.04942 |

* The data were obtained from previous studies conducted by Aldon J. Lusis laboratory[2,3]. Correlations were calculated with the biweight midcorrelation, which is robust to outliers and was used in the studies performed by Aldons J. Lusis laboratory[2]. A negative biweight midcorrelation (bicor) means that the higher plasma thiamine levels in the inbred strains mice is associated with lower levels of the measured trait.
